# Biogeographic Comparison of *Lophelia*-Associated Bacterial Communities in the Western Atlantic Reveals Conserved Core Microbiome

**DOI:** 10.3389/fmicb.2017.00796

**Published:** 2017-05-04

**Authors:** Christina A. Kellogg, Dawn B. Goldsmith, Michael A. Gray

**Affiliations:** St. Petersburg Coastal and Marine Science Center, United States Geological Survey, St. PetersburgFL, USA

**Keywords:** deep-sea corals, microbiome, scleractinian, amplicon sequencing, microbial diversity, Gulf of Mexico

## Abstract

Over the last decade, publications on deep-sea corals have tripled. Most attention has been paid to *Lophelia pertusa*, a globally distributed scleractinian coral that creates critical three-dimensional habitat in the deep ocean. The bacterial community associated with *L. pertusa* has been previously described by a number of studies at sites in the Mediterranean Sea, Norwegian fjords, off Great Britain, and in the Gulf of Mexico (GOM). However, use of different methodologies prevents direct comparisons in most cases. Our objectives were to address intra-regional variation and to identify any conserved bacterial core community. We collected samples from three distinct colonies of *L. pertusa* at each of four locations within the western Atlantic: three sites within the GOM and one off the east coast of the United States. Amplicon libraries of 16S rRNA genes were generated using primers targeting the V4–V5 hypervariable region and 454 pyrosequencing. The dominant phylum was Proteobacteria (75–96%). At the family level, 80–95% of each sample was comprised of five groups: Pirellulaceae, Pseudonocardiaceae, Rhodobacteraceae, Sphingomonadaceae, and unclassified Oceanospirillales. Principal coordinate analysis based on weighted UniFrac distances showed a clear distinction between the GOM and Atlantic samples. Interestingly, the replicate samples from each location did not always cluster together, indicating there is not a strong site-specific influence. The core bacterial community, conserved in 100% of the samples, was dominated by the operational taxonomic units of genera *Novosphingobium* and *Pseudonocardia*, both known degraders of aromatic hydrocarbons. The sequence of another core member, *Propionibacterium*, was also found in prior studies of *L. pertusa* from Norway and Great Britain, suggesting a role as a conserved symbiont. By examining more than 40,000 sequences per sample, we found that GOM samples were dominated by the identified conserved core sequences, whereas open Atlantic samples had a much higher proportion of locally consistent bacteria. Further, predictive functional profiling highlights the potential for the *L. pertusa* microbiome to contribute to chemoautotrophy, nutrient cycling, and antibiotic production.

## Introduction

Over the last decade, the number of publications on deep-sea corals has tripled. This increase in focus has been driven mainly by conservation concerns due to the co-location of many of these cold-water coral habitats with areas where commercial fishing or oil and gas drilling are occurring. These coral habitats form biodiversity hot spots in the deep ocean (Mortensen et al., 1995) and are of interest for a variety of reasons ranging from bioprospecting (Maxwell, 2005), to interpreting paleoclimate conditions using geochemical markers in the coral skeletons (Williams et al., 2006). The lion’s share of attention has been paid to *Lophelia pertusa*, a globally distributed scleractinian coral that creates critical three-dimensional habitat for a large assortment of other invertebrate and vertebrate species (Cordes et al., 2008). This coral species is azooxanthellate, but forms branching colonies that can fuse together to form large mounds. These coral mounds tend to occur on high points in areas of strong current, to facilitate capture-feeding by the coral.

Nutrition is an area of key interest when studying the microbiomes of deep-sea corals. These corals do not have photosynthetic algal partners like tropical corals, so heterotrophic bacterial associates have been hypothesized to play a larger role in nutrient acquisition and cycling (Neulinger et al., 2008; Kellogg et al., 2016; Lawler et al., 2016). A recent study of *L. pertusa* using isotope tracer evidence was able to show nitrogen cycling and transfer of fixed nitrogen and inorganic carbon into the coral tissue (Middelburg et al., 2015). However, the specific microbes involved in the nitrogen cycling remain to be identified.

The bacterial community associated with *L. pertusa* has been described by a number of molecular and culture-based methods (Yakimov et al., 2006; Neulinger et al., 2008, 2009; Hansson et al., 2009; Kellogg et al., 2009; Schöttner et al., 2009; Galkiewicz et al., 2011; Emblem et al., 2012; van Bleijswijk et al., 2015; Meistertzheim et al., 2016). While these studies have been geographically diverse, including the Mediterranean Sea (Yakimov et al., 2006; Meistertzheim et al., 2016), Norwegian fjords (Neulinger et al., 2008, 2009; Schöttner et al., 2009; Emblem et al., 2012), Rockall Banks (Hansson et al., 2009; van Bleijswijk et al., 2015) and the Gulf of Mexico (Kellogg et al., 2009; Galkiewicz et al., 2011), the use of different methodologies prevents direct data comparisons in most cases. A key exception is 16S rRNA gene clone library studies conducted with almost identical methods on *L. pertusa* from the Trondheimsfjord in Norway (Neulinger et al., 2008) and the Gulf of Mexico (Kellogg et al., 2009). Comparing these datasets revealed conserved sequences that were postulated to be *Lophelia*-specific bacterial symbionts (Kellogg et al., 2009).

However, comparisons of these datasets (Neulinger et al., 2008; Kellogg et al., 2009) also indicate that there are differences between the *Lophelia*-associated bacterial communities across the Atlantic. This may be due to the small sample size (less than 1000 16S rRNA gene sequences between the two papers), but could be genuine variation. On a smaller geographic scale, bacterial community differences have been detected between *L. pertusa* colonies from sites as close as 3.9 km in Norway (by T-RFLP profiles; Neulinger et al., 2008) and between sites separated by 36.8 km in the Gulf of Mexico (by 16S rRNA gene clone libraries; Kellogg et al., 2009). Overall, these results support a new paradigm from the shallow-water coral literature: that coral microbiomes consist of three components–(1) a small conserved core of microbial symbionts, (2) a larger group of regionally conserved bacteria specific to a geographic site, depth range, etc., and (3) an environmentally variable bacterial community (Hernandez-Agreda et al., 2016; van de Water et al., 2016).

**FIGURE 1:**
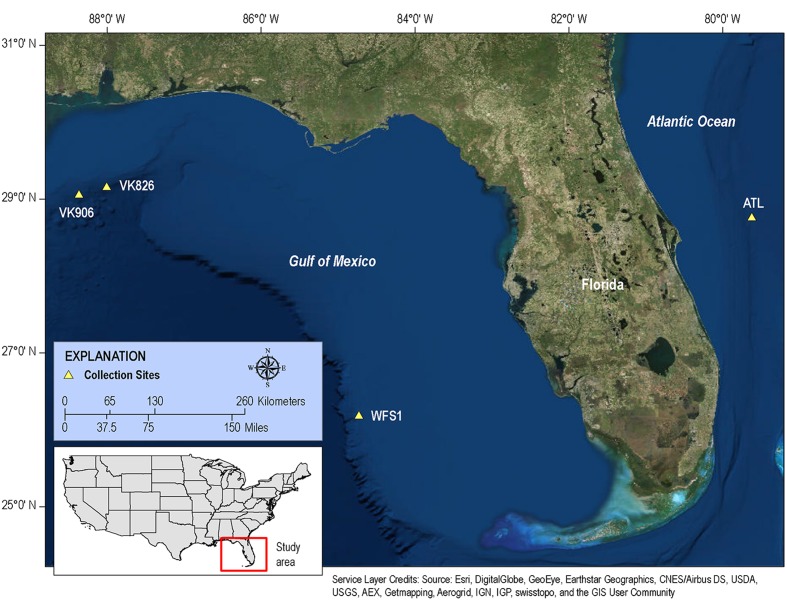
**Map of collection sites.** Samples of *L. pertusa* were collected at four sites in the western Atlantic.

We hypothesize that there is a conserved core of bacterial symbionts held in common by all *L. pertusa*, while the rest of the bacterial community varies depending on local environmental characteristics such as dominant food source, benthic community structure, or water mass parameters. Our objective was to address this issue of intra-regional variation with deep pyrosequencing. To do this, we collected *L. pertusa* from three locations within the Gulf of Mexico and one Atlantic location off the east coast of Florida (**Figure 1**). We analyzed the bacterial communities from three biological replicates (unique coral colonies) at each location in order to examine within-site as well as between-site variation. In an attempt to elucidate the potential functional roles of *L. pertusa*’s bacteria associates, we have employed functional profile prediction (Langille et al., 2013). This technique uses a reference phylogeny of completed bacterial genomes to predict the genomic copy number of each gene family, and those gene families have been linked with experimental evidence of functionality. While the quality of the predictions is directly related to how many relevant environmental bacterial genomes are available in the database, this technique has been increasingly applied to temperate and tropical coral systems (Ainsworth et al., 2015; Morrow et al., 2015; Rubio-Portillo et al., 2016; van de Water et al., 2016; Zaneveld et al., 2016).

## Materials and Methods

### Sample Sites and Collections

Although both orange and white color morphs are common in European waters (Neulinger et al., 2008), only white *L. pertusa* was observed and collected during this study. Three biological replicates (individual colonies of *L. pertusa*) were sampled at each of four sites: Viosca Knoll 906 (VK906), Viosca Knoll 826 (VK826), West Florida Slope 1 (WFS1), and Atlantic 1 (ATL1) (**Figure 1**). Location, temperature, salinity, and depth were recorded for each sample (**Table 1**). There was a depth gradient across the sites, becoming deeper west to east, increasing from 397 to 751 m (**Table 1**). Note that our VK906, the shallowest site, is nearer to a site known was VK862S (CSA International Inc., 2007) and is colloquially known as “Harry’s Reef.” This should not be confused with the VK906 site known as “Robert’s Reef” which has an extensive coral mound (Lunden et al., 2013). Our VK906 site featured isolated colonies of *L. pertusa* with frailer skeletons (“gracilis” morphotype; Brooke et al., 2007). VK826 and WFS1 had extensive thickets of *L. pertusa* with heavily calcified skeletons (“brachycephala” morphotype; Brooke et al., 2007). ATL1 had clusters of *L. pertusa* colonies with several meters of dead coral or rubble between the live corals.

**Table 1:** Sample collection site and associated environmental data for *L. pertusa* coral collections.

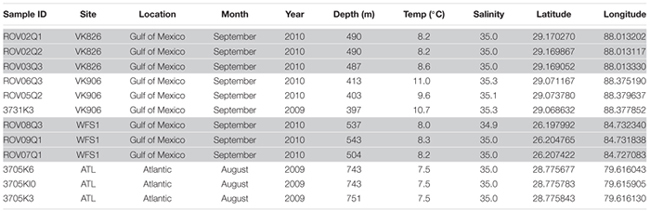

*Shading to show groups of biological replicates from the same site at a glance.*

Samples with names prefixed by 3705 or 3731 were collected using the *Johnson-Sea-Link* submersible (Harbor Branch Oceanographic Institution) using the Kellogg sampler (Kellogg et al., 2009) during cruises in August and September 2009. Briefly, the sampler’s individual compartments were cleaned at the surface using ethanol, filled with sterile deionized water and sealed. Coral branches were collected and placed into the containers after ambient seawater evacuated the freshwater, and then the containers were re-sealed at depth. Samples with names beginning with ROV0 were collected using the remotely-operated vehicle (ROV) *Kraken II* (University of Connecticut) during a research cruise in September 2010. The ROV carried several individual polyvinylchloride (PVC) quivers that were cleaned with ethanol, filled with sterile deionized water and sealed at the surface with rubber stoppers. Immediately prior to collection, a quiver was opened, the sample placed inside, and the quiver sealed before the ROV continued its deployment. Upon return to the surface, all *L. pertusa* samples were transferred to sterile tubes, covered in RNAlater (Life Technologies, Grand Island, NY, USA), and incubated overnight at 4°C to allow the preservative to permeate the coral tissues before transfer to -20°C for long-term storage.

### Nucleic Acid Extraction

All samples from all sites were extracted as follows. The coral fragment was removed from RNAlater using sterile forceps and placed into a sterile aluminum weigh boat in a laminar flow hood. Two polyps from each coral sample (taken from the middle or tip of the branch to avoid any potential contamination at the base where the sampling claw was in contact with the coral) were combined to homogenize the variability of the bacterial community that may exist between polyps (Hansson et al., 2009; Meistertzheim et al., 2016). The calyces containing polyps of *L. pertusa* were broken from the main branch with sterile pliers and placed into separate sterile aluminum dishes by sample. The calyces were cracked open with a sterile hammer and the tissue was removed from the skeleton using an airbrush with sterile phosphate buffered saline (PBS) and sterile forceps, using the minimum volume of PBS necessary per sample (ca. 200–500 μl). While mainly tissue, the samples may have entrained some coral mucus since no specific effort was made to exclude it. DNA was extracted from the samples using the MO BIO PowerPlant DNA Isolation Kit (MO BIO Laboratories; Carlsbad, CA, USA) following the suggested modifications in Sunagawa et al. (2010). Briefly, approximately 50 mg aliquots of the tissue slurry from each sample were processed with the addition of a lysozyme step and additional smaller beads to expedite physical lysis. Three extractions were done per coral sample (for a total of 36 extractions) and then recombined by sample after elution of the DNA from the spin column (resulting in 12 DNA samples, one per coral). The DNA samples were quantified with Quanti-iT PicoGreen dsDNA Assay Kit (Invitrogen; Eugene, OR, USA) per the manufacturer’s protocol.

### 16S rRNA Gene Pyrosequencing

DNA samples were amplified with primers targeting the V4–V5 hypervariable region (563F/926R) of the 16S rRNA gene (Claesson et al., 2010): forward primer (5′ AYTGGGYDTAAAGNG) and reverse primer (5′ CCGTCAATTYYTTTRAGTTT). The forward primer was tagged with one of four MID tags so the samples could be combined for sequencing on three plates. Amplification, pooling and 454 sequencing using GS FLX Titanium chemistry were performed by EnGenCore LLC (Greenville, SC, USA). Sequence data from all samples were deposited in the NCBI Sequence Read Archive (SRA) under Bioproject number PRJNA305617 and are also available online as a USGS data release, https://doi.org/10.5066/F7M32SXM (Kellogg and Goldsmith, 2017).

### Bioinformatics and Statistical Analysis

Analysis of the sequence data was conducted using the bioinformatics packages QIIME 1.8.0 on the Data Intensive Academic Grid (DIAG), a National Science Foundation funded MRI-R2 project #DBI-0959894, and QIIME 1.9.1 on the Amazon Elastic Compute Cloud (Amazon “EC2”) using the QIIME Amazon Machine Image (AMI) (Caporaso et al., 2010b). Our detailed bioinformatics workflow including specific scripts and parameters for each step is included in Supplementary Material.

A total of 1,971,430 raw reads were generated from the 12 individual coral samples. Sequences were screened based on the following quality parameters: sequence length between 200 and 700 bp, minimum average quality score of 25, maximum of one primer mismatch, and maximum of a six homopolymer run (Kunin et al., 2010). The sequences were denoised to reduce the overestimation of operational taxonomic units (OTUs) (Quince et al., 2009; Kunin et al., 2010). This resulted in 1,004,996 sequences total and each individual sample library containing over 40,000 sequences (**Table 2**). An open-reference method with a 97% similarity threshold (Rideout et al., 2014) was used to select OTUs in order to not discard sequences that were not a perfect match to the Greengenes reference database (release 13_8; DeSantis et al., 2006). Chimeras were removed and the OTUs picked using usearch61 (Edgar, 2010). Alignment was done with PyNAST (version 1.2.2) (Caporaso et al., 2010a). Representative sequences from each OTU were selected, assigned a taxonomic classification using uclust (Edgar, 2010), and used to generate a phylogenetic tree (Price et al., 2010). Non-bacterial sequences (i.e., Eukarya, Archaea, chloroplast, mitochondria) and absolute singletons (defined as an OTU present only once in the analysis) were removed from the OTU table. Samples were then randomly rarefied to the size of the smallest library (40,064 sequences) before diversity metrics were calculated (Gihring et al., 2012).

**Table 2:** Alpha diversity analysis of *L. pertusa* coral-associated bacterial communities.

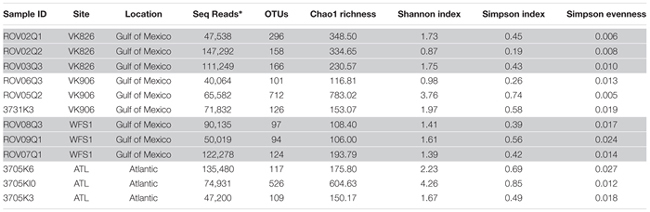

*^∗^Samples were rarefied to 40,064 sequences before diversity metrics were calculated. Shading to show groups of biological replicates from the same site at a glance.*

Phylogenetic Investigation of Communities by Reconstruction of Unobserved States, PICRUSt (Langille et al., 2013) was used to predict the functional gene content of the *L. pertusa*-associated bacterial community by comparing the 16S rRNA amplicon data against a database of existing bacterial genomes. Due to PICRUSt input requirements, sequences were processed a bit differently than as described above for the rest of the QIIME analyses: Raw amplicon reads were scanned with a 5-base sliding window and trimmed when the average Phred quality score within this window dropped below 30 (Ewing et al., 1998). Reads with ambiguous bases, homopolymers greater than 10, or total length less than 100 nucleotides were removed with mothur (Schloss et al., 2009). Chimeric sequences were eliminated with uchime (Edgar et al., 2011). Closed-reference OTUs were picked using QIIME 1.9.1 (Caporaso et al., 2010b) using the Greengenes reference database (release 13_5; DeSantis et al., 2006). Predictive functional profiling of closed-reference OTUs was performed with PICRUSt 1.0.0-dev, basing functional profiles on KEGG Orthology (Kanehisa et al., 2004). Bioinformatic and statistical consulting on the predictive functional profiling was provided by omics2view.consulting GbR (Kiel, Germany).

Alpha and beta diversity calculations as well as relative abundance summaries were done within QIIME 1.9.1 (Caporaso et al., 2010b). **Table 2** contains number of OTUs per sample and alpha diversity metrics including the Chao index (Chao, 1984), Shannon diversity index (Shannon, 1948) and Simpson diversity and evenness indices (Simpson, 1949). To examine differences across samples (beta diversity), three matrices were used based on phylogenetic and taxonomic relationships between sequences. UniFrac measurements were used to evaluate the relative importance of presence/absence of specific taxa within the samples (unweighted UniFrac) vs. the abundance of those taxa (weighted UniFrac) (Lozupone and Knight, 2005). Bray–Curtis dissimilarity was also used to compare differences between each sample based on the number of sequences per OTU. These metrics were visualized via principal coordinate analysis (PCoA) using the vegan package (Oksanen et al., 2016) in R (R Core Team, 2015). Additionally, PRIMER-E (Clarke, 1993) was used to calculate analysis of similarities (ANOSIM) and similarity percentage (SIMPER). Relative abundance bar graphs were prepared in R (R Core Team, 2015) using the ggplot2 package (Wickham, 2009).

The core microbiome was analyzed using QIIME with the minimum fraction of samples set at 100%. The 454 libraries from van Bleijswijk et al. (2015) were screening using the SRA BLAST function (Altschul et al., 1990) to query each library for matches to our core OTU sequences. Full-length 16S rRNA gene sequences from Neulinger et al. (2008) that were taxonomically similar to our core OTUs were trimmed to the V4–V5 region and then aligned against our core OTU sequences to check for similarity using ClustalX (Thompson et al., 1997). FASTA files from the six *L. pertusa* libraries (L1-A, L1-B, L2-A, Lo3-A, Lo3-B, L4-A) available from Meistertzheim et al. (2016)’s Bioproject PRJNA296678 were downloaded and run through the RDP Classifier (Wang et al., 2007) to determine if any bacterial groups similar to our core OTUs were present.

## Results

The four collection sites cover a distance of approximately 1,500 km as the current flows (from Viosca Knoll in the northeastern Gulf of Mexico, south along the West Florida Shelf, around the State of Florida and north into the Atlantic Ocean; **Figure 1**). Sequencing results were obtained from 12 individual *L. pertusa* samples (three biological replicates × four geographic sites). All samples yielded greater than 40,000 reads (**Table 2**), with a range of 94–712 OTUs (mean 219, median 125). Shannon Index diversity values ranged from 0.87 to 4.26 (mean 1.97, median 1.70).

**FIGURE 2:**
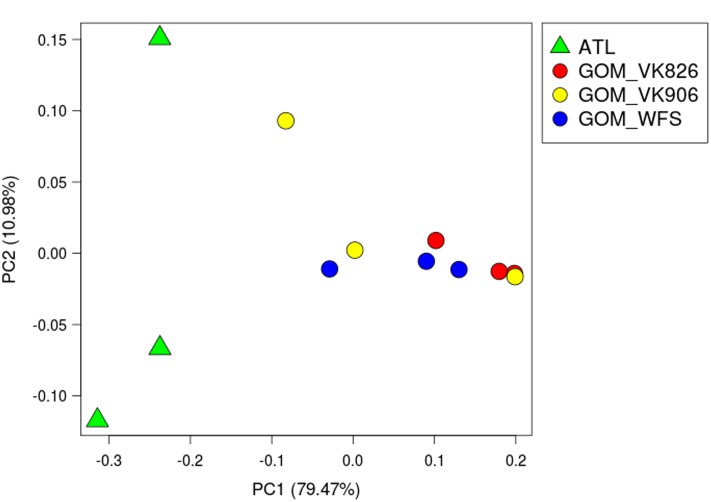
**Principal coordinate analysis (PCoA) plot of weighted UniFrac distance.** Principal coordinates analysis was used to plot beta diversity of coral-associated bacterial communities using the weighted UniFrac matrix. Green triangles indicate Atlantic samples (site ATL). Circles indicate Gulf of Mexico samples: Red–Viosca Knoll 826; Yellow–Viosca Knoll 906; Blue–West Florida Slope 1.

Principal coordinate analysis using weighted UniFrac distances (Lozupone and Knight, 2005) clearly divided the samples by ocean basin, separating the Atlantic from the Gulf of Mexico (**Figure 2**). Using PRIMER-E (Clarke, 1993), we conducted a one-way analysis using Gulf and Atlantic as factors in an Analysis of Similarities (ANOSIM) and found a significant difference between the samples when the data were square-root transformed (*R* = 0.525, *p* = 0.032), but not when the data were 4th-root transformed (*R* = 0.212, *p* = 0.141). This indicates that a difference in abundance of dominant taxa rather than a difference in rare taxa is driving the pattern seen in **Figure 2**. A great deal of within-site variability also was visible, given that biological replicates within a geographic site did not cluster together (**Figure 2**). Similarity percentages calculated using SIMPER on square-root transformed data showed that the average similarity within Gulf samples was 52.45%, with *Novosphingobium* OTU 154189 driving nearly 40% of the similarity. In the Atlantic samples, average similarity was only 38.69, with Oceanospirillales OTU 356942 responsible for 31% and *Novosphingobium* OTU 154189 responsible for 22% of the similarity. These same two OTUs were the highest contributors toward dissimilarity between Atlantic and Gulf samples, but at a much lower level: the average dissimiliarity was 60.62% and these OTUs contributed 8.2 and 7.4%, respectively.

**FIGURE 3:**
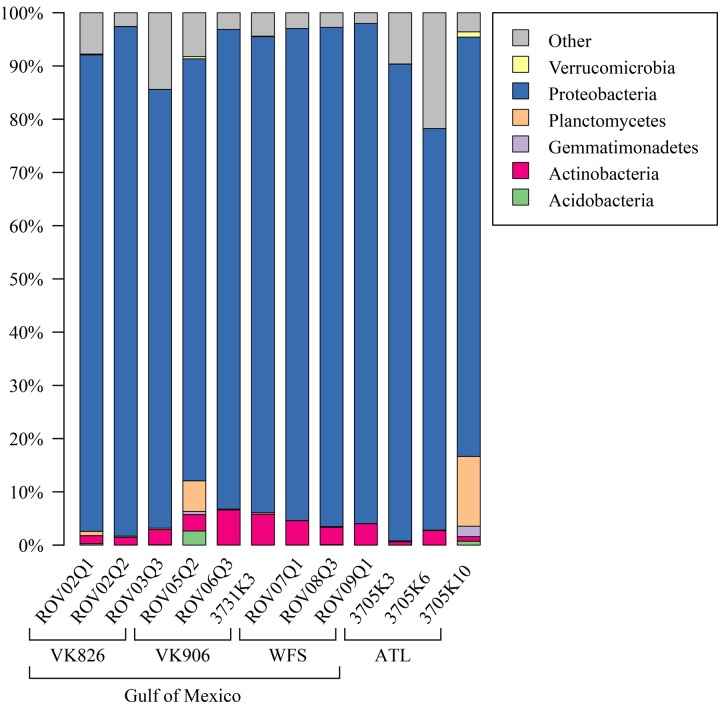
**Relative abundance of bacterial phyla in *L. pertusa* samples.** Bacterial phyla present at ≥1% relative abundance in at least one sample. All remaining taxa are summarized under “Other”.

**FIGURE 4:**
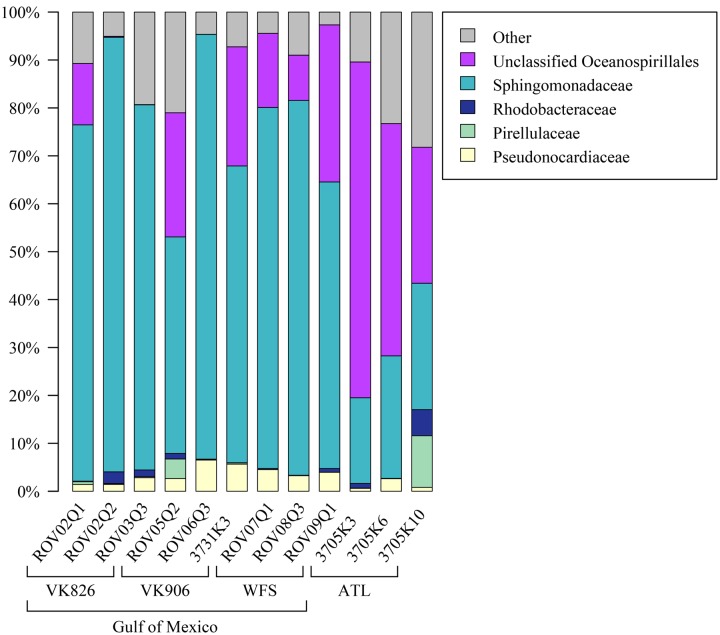
**Relative abundance of families (or the lowest identifiable phylogenetic level) in *L. pertusa* samples.** Bacterial groups that represent ≥1% of the total taxa. All remaining taxa are summarized as “Other”.

Examining the relative abundance at the phylum level (**Figure 3**), all samples were dominated by Proteobacteria (75–96%). Other phyla that made up greater than 1% of total relative abundance included Acidobacteria, Actinobacteria, Gemmatimonadetes, Planctomycetes, and Verrucomicrobia (**Figure 3**). At the family level, the majority of the diversity (70–97% relative abundance) was captured within five families: Pseudonocardiaceae, Pirellulaceae, Rhodobacteraceae, Sphingomonadaceae, and unclassified Oceanospirillales (**Figure 4**). As predicted by the ANOSIM, differences between the Gulf and Atlantic can be seen to be due to differing relative abundances within these taxa (**Figure 4**).

**FIGURE 5:**
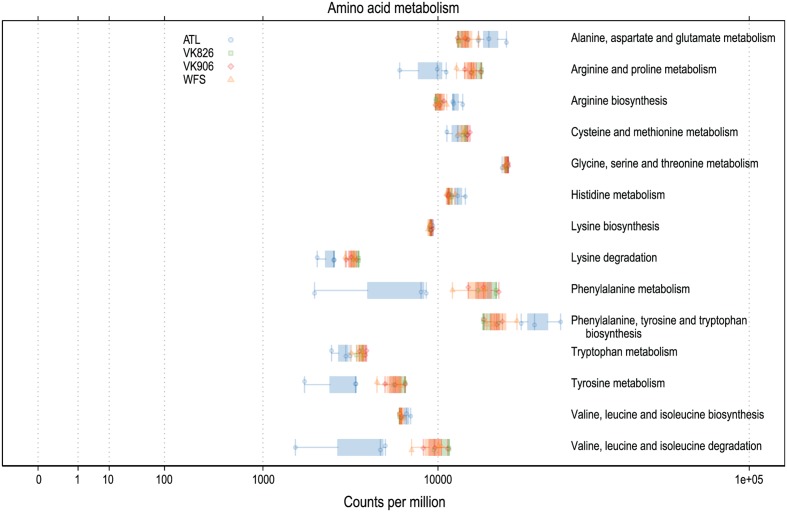
**Predicted amino acid biosynthesis and metabolism capabilities of *L. pertusa*-associated bacterial communities by collection site**.

Functional predictions in a number of other areas suggest high activity on the part of the *L. pertusa*-associated bacterial community. In particular, predicted amino acid metabolism for all samples included the biosynthesis of arginine, isoleucine, leucine, lysine, phenylalanine, tryptophan, tyrosine, and valine (**Figure 5**). Predicted biosynthesis of other secondary metabolites included four antibiotics with three different mechanisms of activity: monobactam (beta lactam), carbapenem (beta lactam), streptomycin (aminoglycoside), and novobiocin (aminocoumarin); in all cases the Atlantic samples were slightly higher than the Gulf of Mexico samples (Supplementary Files). Predicted energy metabolism hinted at chemoautotrophy and nutrient cycling with all samples showing high values for carbon fixation, methane metabolism, nitrogen metabolism, oxidative phosphorylation, and sulfur metabolism (Supplementary Files). Additional predictive profiling data showing functional pathway abundance and completeness by site and by phylum are included in the Supplementary Files.

**FIGURE 6:**
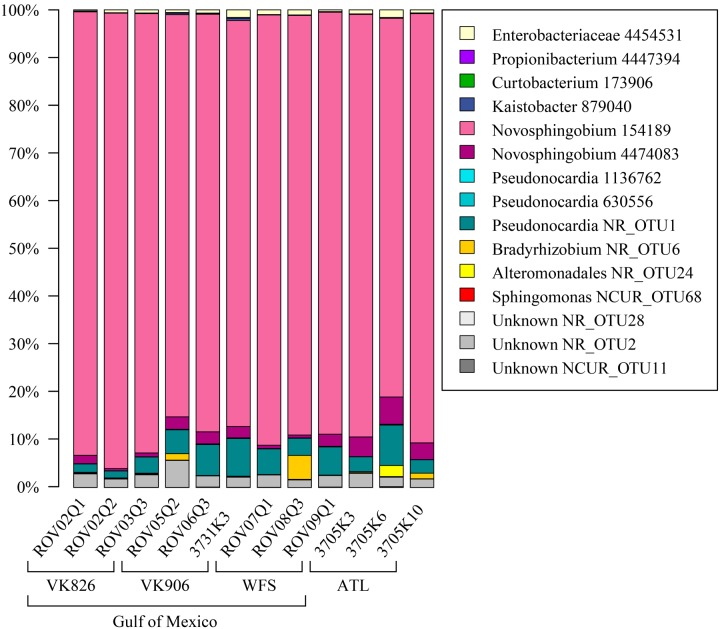
**Conserved total core bacterial OTUs in *L. pertusa.*** Relative abundance of the 15 OTUs found to be conserved across all 12 coral samples.

**FIGURE 7:**
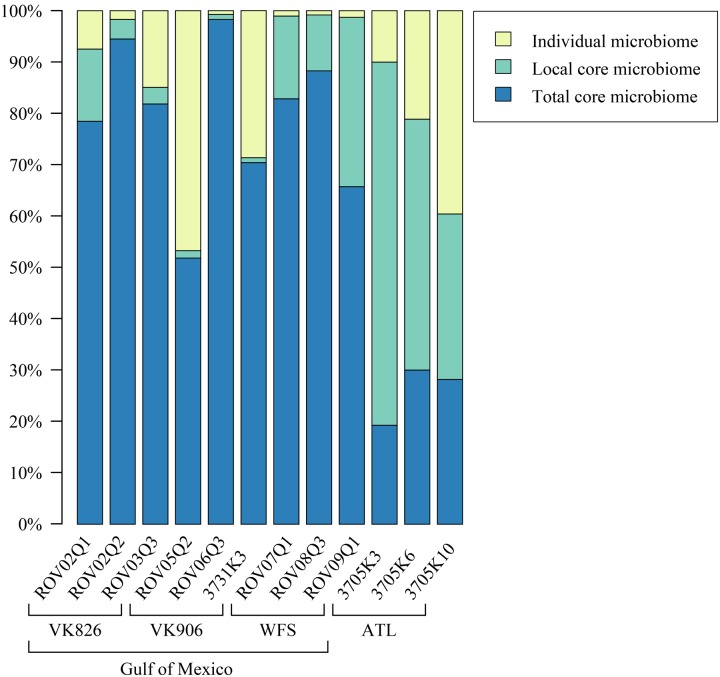
**Relative abundance of total core (OTUs found in all samples), local core (OTUs found in all samples from one geographic site), and remaining variable OTUs present in each coral colony**.

**FIGURE 8:**
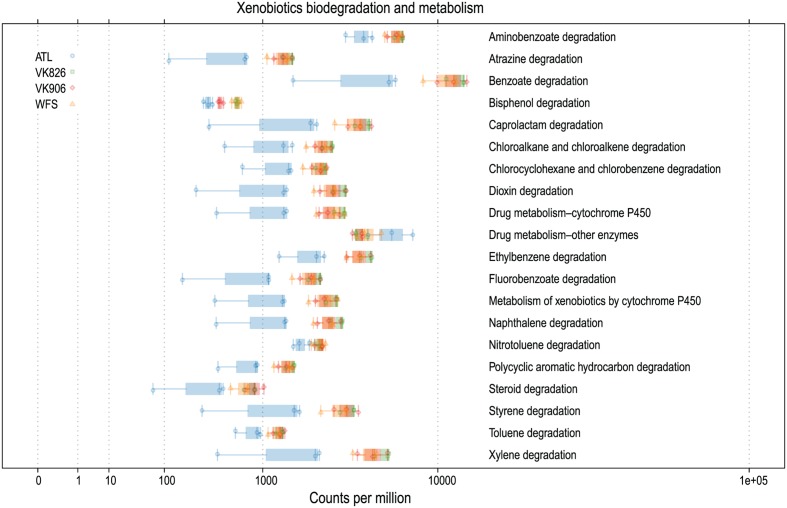
**Predicted xenobiotics degradation and metabolism capabilities of *L. pertusa*-associated bacterial communities by collection site**.

Sequences were examined to determine if a conserved core bacterial community (Shade and Handelsman, 2012) exists for *L. pertusa*, here defined as OTUs present in all 12 samples (100% inclusion). Fifteen OTUs were identified as being held in common (**Figure 6**), with the core being dominated by an OTU (154189) from the genus *Novosphingobium*. A second *Novosphingobium* OTU and three OTUs from the genus *Pseudonocardia* are the next most prominent taxa. This is striking given that both these bacterial genera are known for their ability to degrade aromatic compounds including hydrocarbons (Notomista et al., 2011; Huang and Goodfellow, 2012). Also present at lower relative abundance were three OTUs that could not be classified, and one OTU each of Alteromonadales, Enterobacteriaceae, and genera *Bradyrhizobium*, *Curtobacterium*, *Kaistobacter*, *Propionibacterium*, and *Sphingomonas* (**Figure 6**). The total core taxa cumulatively make up 19–95% of the total relative abundance (**Figure 7**), with a clear difference between the three ATL samples (19–30%) and the nine Gulf of Mexico samples (52–95%). Samples from the Atlantic are dominated by a local core (sequences conserved in samples from that site) compared to total core (**Figure 7**). The lower proportion of *Novosphingobium* and *Pseudonocardia* in ATL samples (**Figures 4**, **7**) is likely the driver behind lower functional predictions of xenobiotic biodegradation and metabolism for those samples vs. Gulf of Mexico samples (**Figure 8**).

## Discussion

Studies on the stability of tropical coral-associated bacterial communities have found that temporal (Hong et al., 2009; Ceh et al., 2011) and geographic (Guppy and Bythell, 2006; Klaus et al., 2007; Hong et al., 2009; Littman et al., 2009; Kvennefors et al., 2010; McKew et al., 2012; Morrow et al., 2012) effects do occur, but that species-specificity can still be detected (Rohwer et al., 2002; Bourne and Munn, 2005; Hong et al., 2009; Kvennefors et al., 2010; McKew et al., 2012; Morrow et al., 2012). All of our samples were collected at the same time of year (August–September), so it is unlikely that we would detect any temporal differences (e.g., linked to surface bloom dynamics). The temperature variation observed across our locations was 7.5–11.0°C and this was considered likely to be a primary environmental variable; however, no significant patterns were observed. Similarly, the depth range from 397 to 751 m, while potentially a proxy for other environmental gradients, also did not yield any significant patterns. A population study of *L. pertusa* found genetic differentiation between corals in the Gulf of Mexico (including our Viosca Knoll sites) and those off the Southeastern United States (including Cape Canaveral collections that overlap our ATL site) (Morrison et al., 2011). The host populations’ genetic differences could be linked to the differences seen in the microbiomes between the Gulf and Atlantic sites (**Figure 2**). Additionally, since our samples included both tissue and mucus bacterial communities, it is possible that some of the inter-sample differences seen (i.e., within a site), reflect different levels of mucus entrainment in each sample.

The mucus of *L. pertusa* has been shown to include 14 of the 20 main amino acids: alanine, arginine, aspartic acid, glutamic acid, glycine, histidine, isoleucine, leucine, lysine, phenylalanine, serine, threonine, tyrosine, valine (Wagner et al., 2011). Cultured fungi from *L. pertusa* have already been shown to metabolize 6 of these, in addition mucus carbohydrates glucose, mannose, *N*-acetyl glucosamine, galactose, xylose, and fucose (Galkiewicz et al., 2012). Predictive functional analysis suggests the bacterial community is capable of metabolizing all 14 of these amino acids (**Figure 5**) and many carbohydrates, including amino sugars, nucleotide sugars, fructose, mannose, galactose, and sucrose (Supplementary Files). This suggests the possibility of the microbiome recycling carbon and nitrogen from the coral’s mucus.

In addition to capture-feeding on particulates, *L. pertusa* has been shown to be an opportunist feeder capable of taking up dissolved free amino acids (Gori et al., 2013; Mueller et al., 2014). This matches well with the bacterial community’s above mentioned capacity to metabolize a wide range of amino acids (**Figure 5**). Functional predictions also suggest that the *L. pertusa* bacterial community is capable of synthesizing arginine, tyrosine, and 6 of the 9 putatively “essential” amino acids (isoleucine, leucine, lysine, phenylalanine, tryptophan, valine; **Figure 5**). This, in addition to a high predicted capacity for fatty acid biosynthesis (Supplementary Files), supports recent findings using stable isotopes to prove transfer of fixed nitrogen and inorganic carbon into these tissue components (Middelburg et al., 2015). The chemoautotrophy and nitrogen cycling observed by Middelburg et al. (2015) is further echoed by the high predictive values for energy metabolism by the bacterial community, including carbon fixation, oxidative phosphorylation, and metabolism of methane, nitrogen, and sulfur (Supplementary Files). While the contribution of this chemosynthesis appears to serve only a minor part of *L. pertusa*’s metabolic requirements, it may be important enough to shape the bacterial community, resulting in conservation of either specific taxonomic or functional groups.

The Atlantic and Gulf samples were dominated by the same four families and one unclassified order (**Figure 4**), but with significant differences in relative abundance. The Gulf samples were more enriched in Sphingomonadaceae and Pseudonocardiaceae, whereas the Atlantic samples were dominated by unclassified Oceanospirillales. This translated to Gulf samples containing a higher proportion of the conserved total core, including *Novosphingobium* and *Pseudonocardia* OTUs compared to the Atlantic (**Figures 6**, **7**). Both these genera are known to be able to degrade aromatic hydrocarbons (e.g., Notomista et al., 2011; Huang and Goodfellow, 2012). We acknowledge that aromatic hydrocarbon degrading bacteria in general are virtually ubiquitous (e.g., Hazen et al., 2010; Kostka et al., 2011; Ghosal et al., 2016) and their presence is not necessarily specifically related to the presence of hydrocarbons (although presence of hydrocarbons can enrich for degraders; Hazen et al., 2010). However, given that the Gulf of Mexico has many natural hydrocarbon seeps (MacDonald et al., 1990; Roberts et al., 2007) and localized hydrocarbon seepage was observed at Viosca Knoll 826 (Cordes et al., 2006), it is possible that *L. pertusa* colonies in the Gulf maintain a higher abundance of core bacteria that are capable of breaking down these carbon sources (**Figure 8**). For comparison, neither *Novosphingobium* nor *Pseudonocardia* were detected in the microbiomes of four other species of deep-sea corals in the Atlantic (Kellogg et al., 2016; Lawler et al., 2016). However, previous research does not indicate incorporation of seep carbon into these corals (Becker et al., 2009), implying that bacterial action may be to detoxify rather than to provide nutrition.

*Pseudonocardia* are also known to be antibiotic producers and so another possibility for their core function could be to protect the coral from invading pathogens or to structure the microbiome by preventing overgrowth of certain taxa (Zhang et al., 2013). All samples showed relatively high predicted values for production of a number of antibiotics: monobactam, carbapenem, streptomycin, and novobiocin, but in all cases the Atlantic samples were slightly higher (Supplementary Files). While several Gulf of Mexico samples had higher relative abundance of Pseudonocardiaceae (**Figure 4**), Atlantic samples also contained other actinobacterial species (*Actinomyces* and *Microbacterium*) in their local or individual microbiomes (**Figure 7**) at less than 1% total relative abundance.

The dominant bacterial phylum detected in *L. pertusa* was Proteobacteria, which corroborates prior work conducted using clone libraries (Neulinger et al., 2008; Kellogg et al., 2009) as well as pyrosequencing targeting the V1–V3 (Meistertzheim et al., 2016) and V4 (van Bleijswijk et al., 2015) variable regions of the 16S rRNA gene. Additional major phyla seen in our dataset have also been previously detected in *L. pertusa*, but not consistently by all studies; i.e., Acidobacteria (Yakimov et al., 2006; van Bleijswijk et al., 2015; Meistertzheim et al., 2016), Actinobacteria (Neulinger et al., 2008; Galkiewicz et al., 2011; Meistertzheim et al., 2016), Gemmatimonadetes (van Bleijswijk et al., 2015), Planctomycetes (Neulinger et al., 2008; Kellogg et al., 2009; van Bleijswijk et al., 2015; Meistertzheim et al., 2016), and Verrucomicrobia (Neulinger et al., 2008; van Bleijswijk et al., 2015).

Prior clone library and *in situ* hybridization studies identified mycoplasmas as a component of the *L. pertusa* microbiome (Neulinger et al., 2008, 2009; Kellogg et al., 2009), however, neither of the recent pyrosequencing efforts on this coral detected them (van Bleijswijk et al., 2015; Meistertzheim et al., 2016). Our hypotheses were that (a) since van Bleijswijk et al. (2015) only examined mucus, and it was previously shown by fluorescent *in situ* hybridization (FISH) that the mycoplamas were in the tissue component (Neulinger et al., 2009), that explained their absence in one dataset, and (b) perhaps the low number of sequences examined (446 per sample) by Meistertzheim et al. (2016) was insufficient to detect mycoplasmas. With our greater sequencing depth, we did observe Tenericutes in 7 of 12 samples, but always at less than 1% relative abundance. Of those samples, 5 contained sequences that were identified to the genus *Mycoplasma* (ROV02Q1, 3731K3, ROV07Q1, 3705K3, 3705K10), including at least one replicate from each geographic site. This suggests that while present in this coral, there could be either strong positive selection for mycoplasmal sequences using the clone library protocol (e.g., Kellogg et al., 2009; Gray et al., 2011), or conversely, that there may be negative selection against mycoplasmal sequences in pyrosequencing methods. An alternative (and not mutually exclusive) hypothesis put forward by Meistertzheim et al. (2016) is that bacterial community variations seen between and within *L. pertusa* colonies are due to sample libraries being dominated by the microbiome of the gut cavity and therefore reflective of the varied and opportunistic diet of the host. Interestingly, when the *Mycoplasma* OTU from this study was queried using BLAST (Altschul et al., 1990), it did not closely match to mycoplasmal clones from either the Kellogg et al. (2009) or Neulinger et al. (2008) studies. Instead, it was 92% similar to mycoplasmal clones from deep-sea bamboo corals (Penn et al., 2006) and 91% similar to a mycoplasmal clone from the Aleutian gorgonian *Cryogorgia koolsae* (Gray et al., 2011). This was unexpected because Gray et al. (2011) had constructed a phylogenetic tree based on clone library sequences showing that there were two major clades of coral-associated mycoplasma; one that included gorgonian-associated sequences and one that only included *L. pertusa*-associated sequences.

*Propionibacterium* OTU 4447394 makes up a small portion of the conserved core identified in these *L. pertusa* samples. However, because *Propionibacterium* has been recently identified as a rare but conserved member of shallow and mesophotic tropical corals (Ainsworth et al., 2015; Hester et al., 2016), we investigated it more closely. We found that OTU 4447394 had 99% identity with 100% coverage against two *Propionibacterium* clones from Norwegian fjord *L. pertusa* (Sequence IDs: AM911348, AM911422; Neulinger et al., 2008) and also against three reads from *L. pertusa* mucus collected on Rockall Bank in the UK (Sequence IDs: SRA:ERR951478.4168.1, SRA:ERR951478.3202.1, SRA:ERR951478.1453.1; van Bleijswijk et al., 2015). This shows that *Propionibacterium* OTU 4447394 is present in *L. pertusa* datasets on both sides of the Atlantic. We used BLAST (Altschul et al., 1990) to search this OTU against other deep-sea coral sequences derived using the V4 region, confirming this same sequence in the core of *Paramuricea placomus* (Kellogg et al., 2016) and in the core of *Anthothela* sp. (Lawler et al., 2016). In all cases, there was a difference of three single nucleotide insertions (duplications of the prior base) in OTU 4447394 compared to the sequences found outside this study. We were unable to directly compare against 454 sequences from Mediterranean *L. pertusa* (Meistertzheim et al., 2016) because of differing primer regions. However, when we screened the six *L. pertusa* libraries available from the Meistertzheim et al. (2016) study by running them through RDP Classifier, 5 of the 6 libraries contained the genus *Propionibacterium*.

Ainsworth et al. (2015) used FISH to show that *Propionibacterium* cells were located intracellularly within the photosynthetic dinoflagellates housed by the tropical corals. However, there are no photosynthetic symbionts in *L. pertusa*, *P. placomus*, or *Anthothela* sp. given that these corals were all collected at greater than 300 m depth, living in darkness. It would be illuminating to conduct similar FISH studies to determine the location of *Propionibacterium* in deep-sea corals. Further, it would be helpful to be able to compare full-length 16S rRNA sequences (or better yet whole genomes) from *Propionibacterium* in these deep-sea corals against those in photosynthetic symbiont-bearing corals to determine what roles these apparently ubiquitous symbionts are playing. That said, without FISH or culture-based studies, we cannot absolutely rule out the possibility that these *Propionibacterium* sequences are contaminants from DNA extraction kits (Salter et al., 2014).

In addition to *Propionibacterium*, the Mediterranean *L. pertusa* libraries (Meistertzheim et al., 2016) contained several of the other taxa identified as core OTUs in our study. Four libraries contained Alteromonadales (L1-A, Lo3-A, Lo3-B, and L4-A), three libraries contained Enterobacteriaceae (L1-A, L1-B, and L2-A), one library contained *Pseudonocardia* (L1-B) and one library contained *Sphingomonas* (L1-A). However, no counterpart was observed in the Mediterranean libraries to our most relatively abundant core OTU, *Novosphingobium* 154189.

We used BLAST (Altschul et al., 1990) to assess whether any of our core OTUs showed significant sequence matches to other coral-associated studies. Alteromonadales OTU NR_OTU24 was 96% similar to a clone from coral *Tubastraea coccinea* (Sequence ID: JF925023.1; Yang et al., 2013) and four clones from a deep-sea black coral (Sequence IDs: DQ395570.1, DQ395572.1, DQ395576.1, DQ395594.1; Penn et al., 2006). Both *Novosphingobium* total core OTUs in this study were 98% identical to three deep seawater clones collected near deep-sea corals (Sequence IDs: DQ396195, DQ396202, DQ396285; Penn et al., 2006). Since it is impossible to collect coral samples without exposing them to local seawater, there always exists the possibility that we could detect marine microorganisms. However, coral microbiome samples have consistently been shown to be very different from the bacterial community of the surrounding seawater (e.g., Yakimov et al., 2006; Neulinger et al., 2008; van Bleijswijk et al., 2015) and we would be surprised to find a transient marine microbial signal not overwhelmed by the signal from the tissue community.

A main goal of this study was to determine and characterize the conserved core bacterial community of *L. pertusa*. However, without having samples from eastern Atlantic and Pacific coral colonies that were processed the same way and sequenced to the same depth, we cannot be certain that the identified core is maintained outside the western Atlantic. For example, *Novosphingobium* dominated our core and was not detected in other *L. pertusa* datasets. However, the consistent identification of *Propionibacterium* in all three next-generation *L. pertusa* 16S rRNA datasets (this study; van Bleijswijk et al., 2015; Meistertzheim et al., 2016), much like the prior sequence matches between Gulf of Mexico and Norwegian clone library studies (Neulinger et al., 2008; Kellogg et al., 2009) suggests that there are some bacterial phylotypes that are conserved by this coral regardless of geographic provenance.

## Conclusion

Our data show that *L. pertusa* coral colonies in the western Atlantic have a conserved core microbiome of 15 OTUs, dominated by the genera *Novosphingobium* and *Pseudonocardia*. Regional differences were observed between bacterial communities inside the Gulf of Mexico and those in the open Atlantic. Gulf sample communities were primarily composed of the conserved core vs. Atlantic samples that were dominated by locally consistent bacteria not present in the conserved core. Given the dominance of aromatic-hydrocarbon degrading species in the conserved core, we hypothesize that this selection may be driven by the common presence of natural hydrocarbon seepage in the Gulf of Mexico. Mycoplasmal associates previously identified in *L. pertusa* by clone libraries were not detected. However, a member of the conserved core identified in this study, *Propionibacterium* OTU 4447394, was matched to sequences derived from *L. pertusa* in Norway and off Great Britain suggesting a role as a geographically conserved symbiont. Functional predictions generated by PICRUSt align with recent discoveries of chemoautotrophy and nutrient cycling in *L. pertusa*, highlighting roles of the bacterial community in amino acid biosynthesis and metabolism, carbon fixation, and metabolism of methane, nitrogen, and sulfur.

## Author Contributions

CK planned the experimental design, conducted data analysis and wrote the manuscript. DG conducted data analysis and formatted figures. MG performed the DNA extractions and provided text for the Materials and Methods section. All authors edited the manuscript.

## Conflict of Interest Statement

The authors declare that the research was conducted in the absence of any commercial or financial relationships that could be construed as a potential conflict of interest.
