# Supplementary material for: Biogeographic Comparison of Lophelia-Associated Bacterial Communities in the Western Atlantic Reveals Conserved Core Microbiome
Source: Front Microbiol. 2017 May 4;8:796. doi: 10.3389/fmicb.2017.00796 (PMC5415624; doi:10.3389/fmicb.2017.00796)
Supplement: Supplementary file 1 [file Data_Sheet_1.ZIP › Lophelia_supplementary_material/ SupplementaryMaterial_Lophelia.docx]

Supplementary Material

Biogeographic comparison of *Lophelia*-associated bacterial communities in the western Atlantic reveals conserved core microbiome

Christina A. Kellogg*, Dawn B. Goldsmith, Michael A. Gray

*** Correspondence:** Christina Kellogg: ckellogg@usgs.gov

# Supplementary Material

Detailed workflow of QIIME scripts, including comments and screen outputs is presented in file **Workflow.txt**

Pathway abundance by site (VK826, VK906, WFS, ATL) for amino acid metabolism, biosynthesis of other secondary metabolites, carbohydrate metabolism, cell growth and death, cell motility, energy metabolism, folding/sorting/degradation, glycan biosynthesis and metabolism, lipid metabolism, membrane transport, metabolism of cofactors and vitamins, metabolism of other amino acids, metabolism of terpenoids and polyketides, nucleotide metabolism, replication and repair, signal transduction, transcription, translation, and xenobiotics biodegradation and metabolism is presented in file **Lophelia_pathway_abundance_amplicon.pdf**

Pathway completeness scores by site (VK826, VK906, WFS, ATL) for amino acid metabolism, biosynthesis of other secondary metabolites, carbohydrate metabolism, cell growth and death, cell motility, energy metabolism, folding/sorting/degradation, glycan biosynthesis and metabolism, lipid metabolism, membrane transport, metabolism of cofactors and vitamins, metabolism of other amino acids, metabolism of terpenoids and polyketides, nucleotide metabolism, replication and repair, signal transduction, transcription, translation, and xenobiotics biodegradation and metabolism are presented in file **Lophelia_pathway_completeness_amplicon.pdf**

For functional pathways with a completeness score ≥ 75%, pathway abundance is broken down by site (VK826, VK906, WFS, ATL) in file **Lophelia_pathway_phylum_amplicon.pdf**
