# Supplementary material for: Biogeographic Comparison of Lophelia-Associated Bacterial Communities in the Western Atlantic Reveals Conserved Core Microbiome
Source: Front Microbiol. 2017 May 4;8:796. doi: 10.3389/fmicb.2017.00796 (PMC5415624; doi:10.3389/fmicb.2017.00796)
Supplement: Supplementary file 1 [file Data_Sheet_1.ZIP › Lophelia_supplementary_material/Lophelia_pathway_completeness_amplicon.pdf]

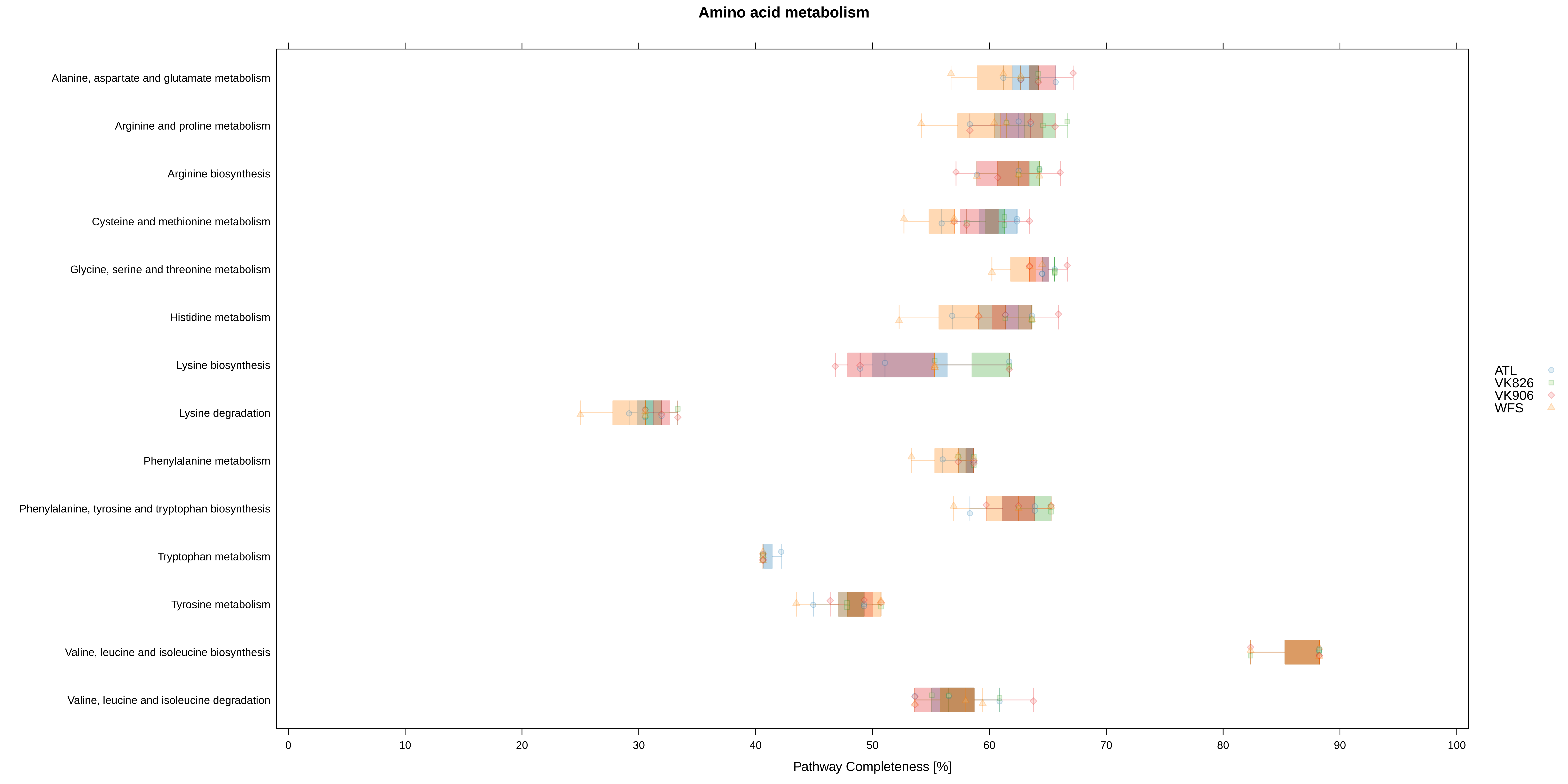
Amino acid metabolism
Alanine, aspartate and glutamate metabolism
Arginine and proline metabolism
Arginine biosynthesis
Cysteine and methionine metabolism
Glycine, serine and threonine metabolism
Histidine metabolism
Lysine biosynthesis
Lysine degradation
Phenylalanine metabolism
Phenylalanine, tyrosine and tryptophan biosynthesis
Tryptophan metabolism
Tyrosine metabolism
Valine, leucine and isoleucine biosynthesis
Valine, leucine and isoleucine degradation
0
10
20
30
40
50
60
70
80
90
100
Pathway Completeness [%]
ATL
VK826
VK906
WFS

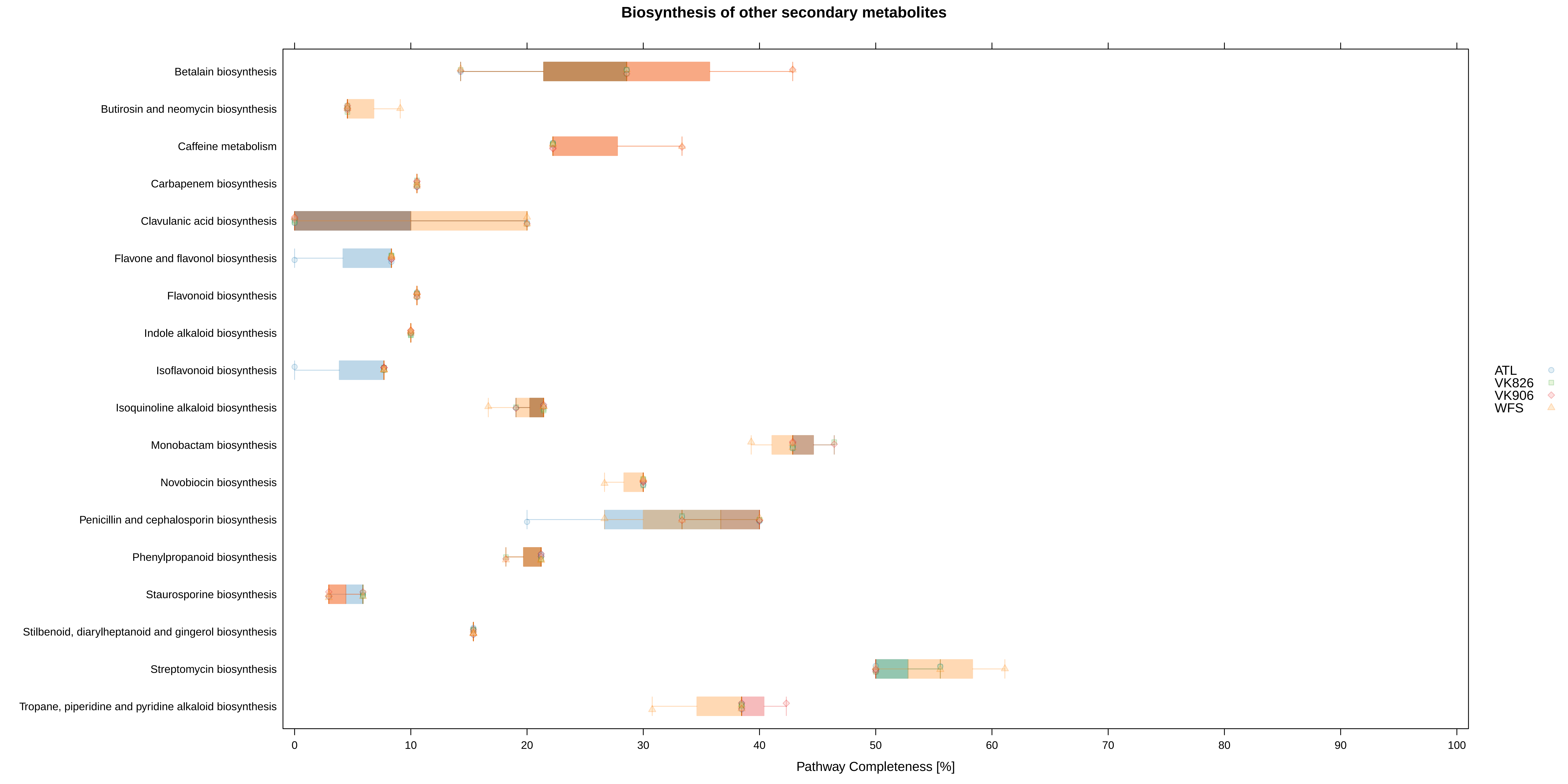
Biosynthesis of other secondary metabolites
Betalain biosynthesis
Butirosin and neomycin biosynthesis
Caffeine metabolism
Carbapenem biosynthesis
Clavulanic acid biosynthesis
Flavone and flavonol biosynthesis
Flavonoid biosynthesis
Indole alkaloid biosynthesis
Isoflavonoid biosynthesis
Isoquinoline alkaloid biosynthesis
Monobactam biosynthesis
Novobiocin biosynthesis
Penicillin and cephalosporin biosynthesis
Phenylpropanoid biosynthesis
Staurosporine biosynthesis
Stilbenoid, diarylheptanoid and gingerol biosynthesis
Streptomycin biosynthesis
Tropane, piperidine and pyridine alkaloid biosynthesis
0
10
20
30
40
50
60
70
80
90
100
Pathway Completeness [%]
ATL
VK826
VK906
WFS

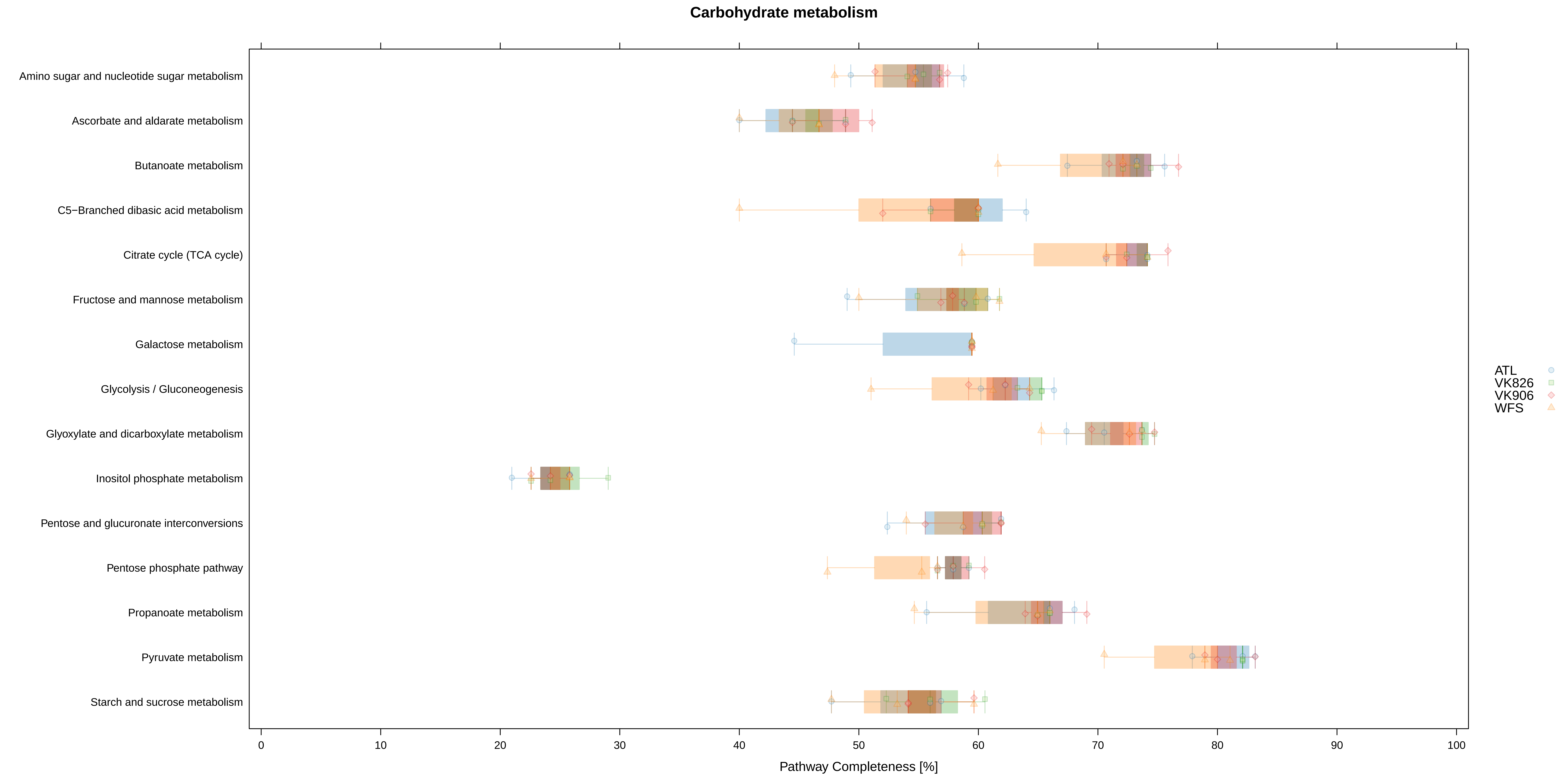

Carbohydrate metabolism
Amino sugar and nucleotide sugar metabolism
Ascorbate and aldarate metabolism
Butanoate metabolism
C5−Branched dibasic acid metabolism
Citrate cycle (TCA cycle)
Fructose and mannose metabolism
Galactose metabolism
Glycolysis / Gluconeogenesis
Glyoxylate and dicarboxylate metabolism
Inositol phosphate metabolism
Pentose and glucuronate interconversions
Pentose phosphate pathway
Propanoate metabolism
Pyruvate metabolism
Starch and sucrose metabolism
0
10
20
30
40
50
60
70
80
90
100
Pathway Completeness [%]
ATL
VK826
VK906
WFS

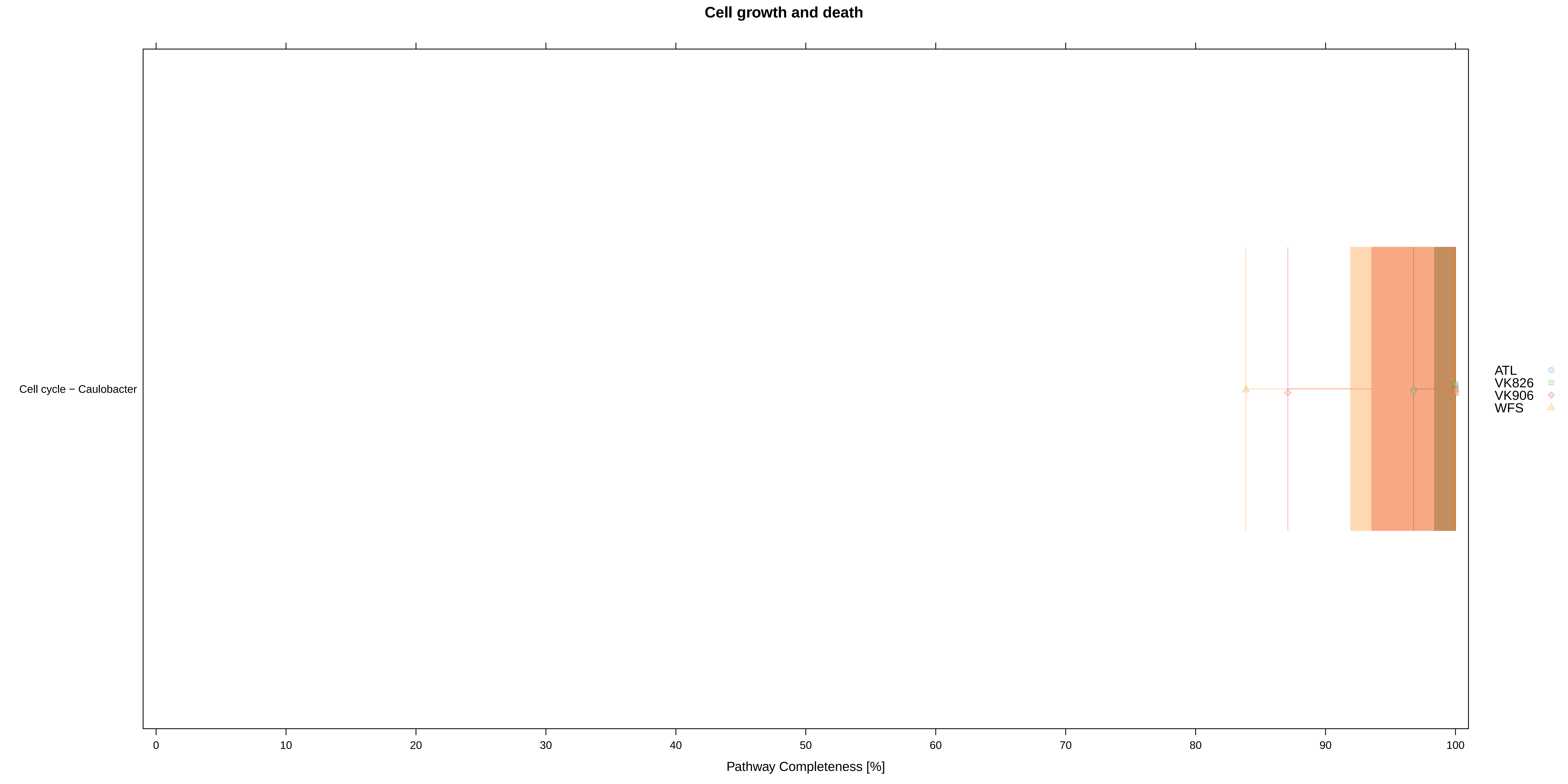
Cell growth and death
Cell cycle − Caulobacter
0
10
20
30
40
50
60
70
80
90
100
Pathway Completeness [%]
ATL
VK826
VK906
WFS

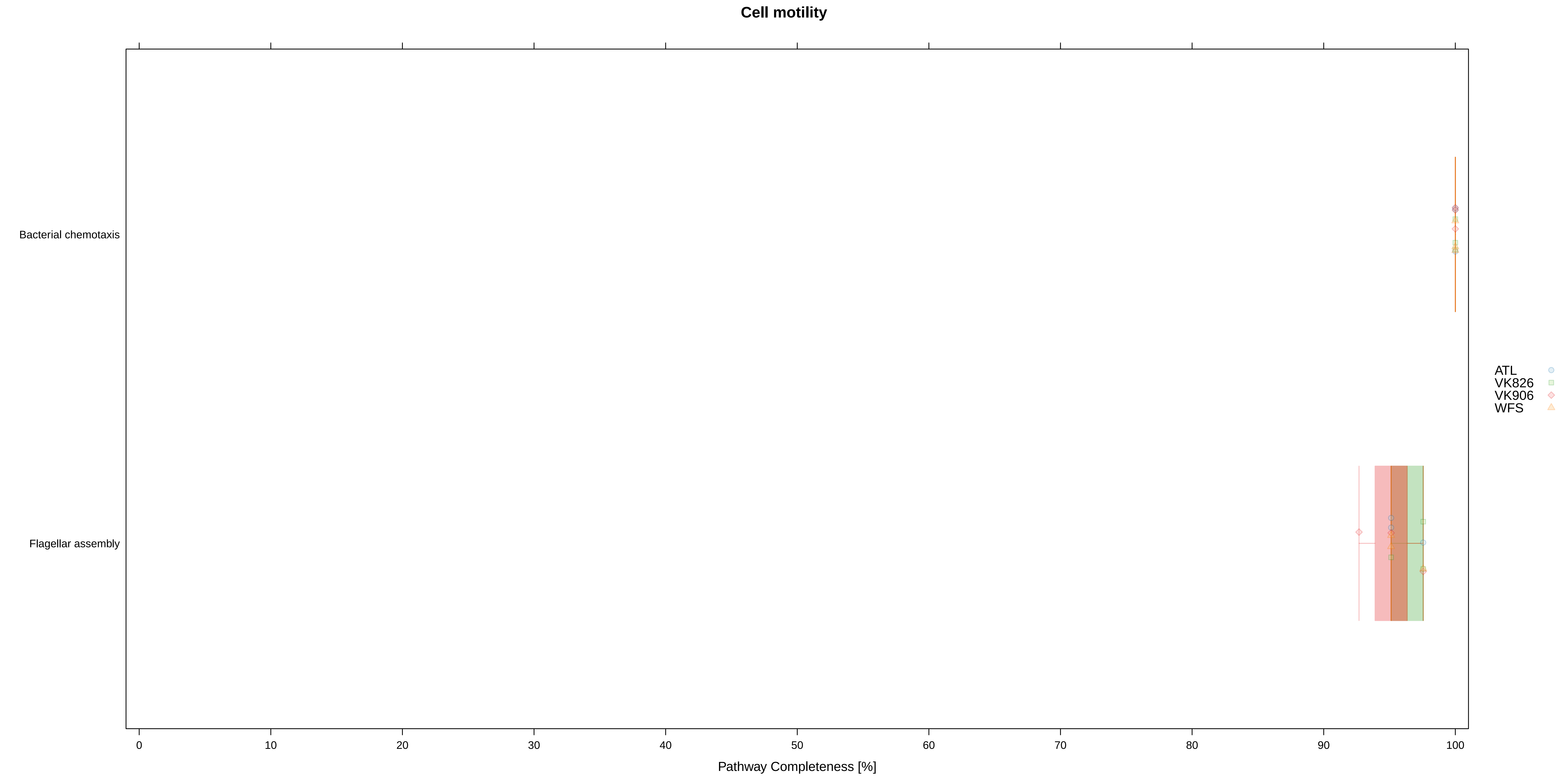
Cell motility
Bacterial chemotaxis
Flagellar assembly
0
10
20
30
40
50
60
70
80
90
100
Pathway Completeness [%]
ATL
VK826
VK906
WFS

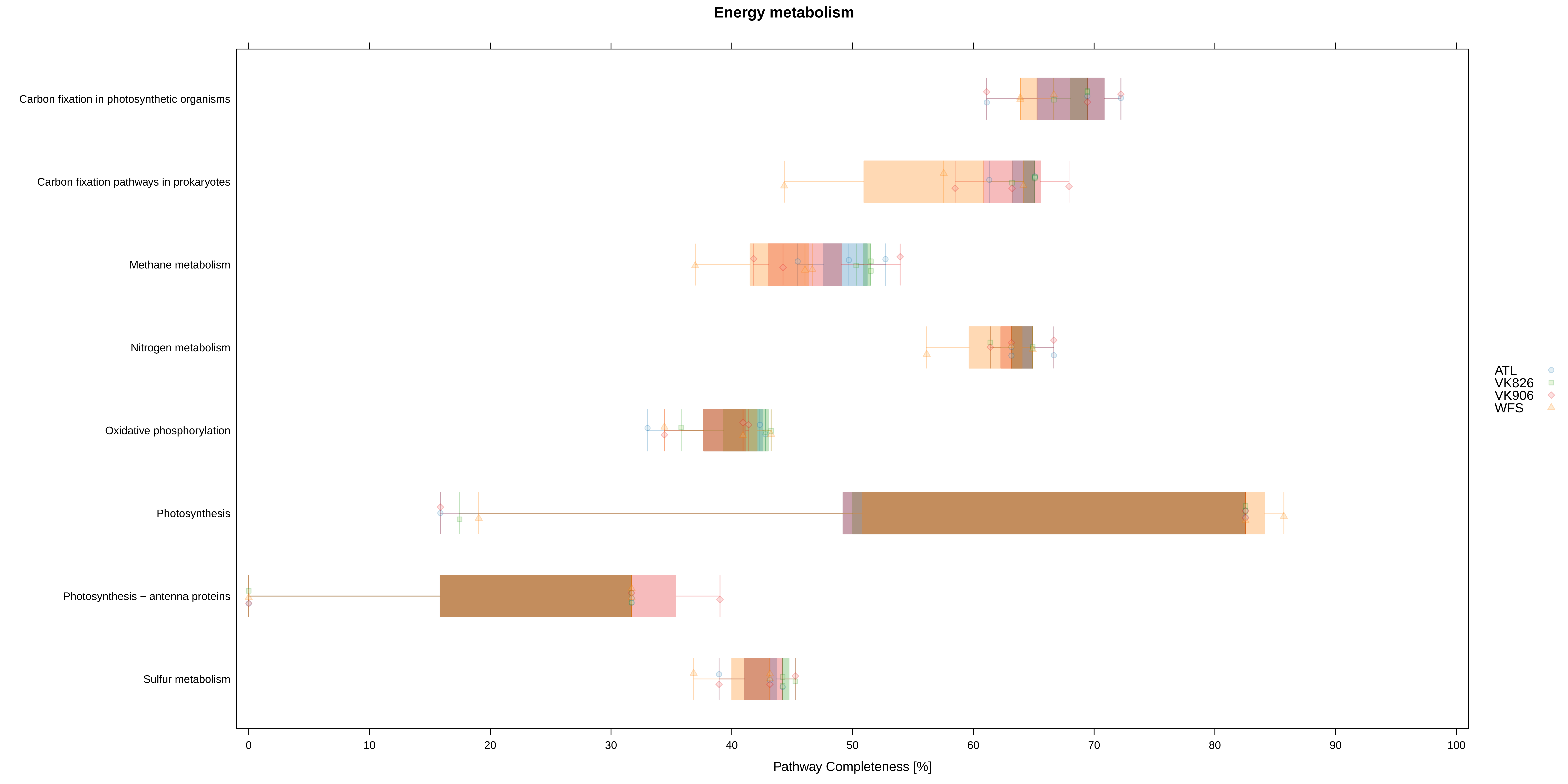

Energy metabolism
Carbon fixation in photosynthetic organisms
Carbon fixation pathways in prokaryotes
Methane metabolism
Nitrogen metabolism
Oxidative phosphorylation
Photosynthesis
Photosynthesis − antenna proteins
Sulfur metabolism
0
10
20
30
40
50
60
70
80
90
100
Pathway Completeness [%]
ATL
VK826
VK906
WFS

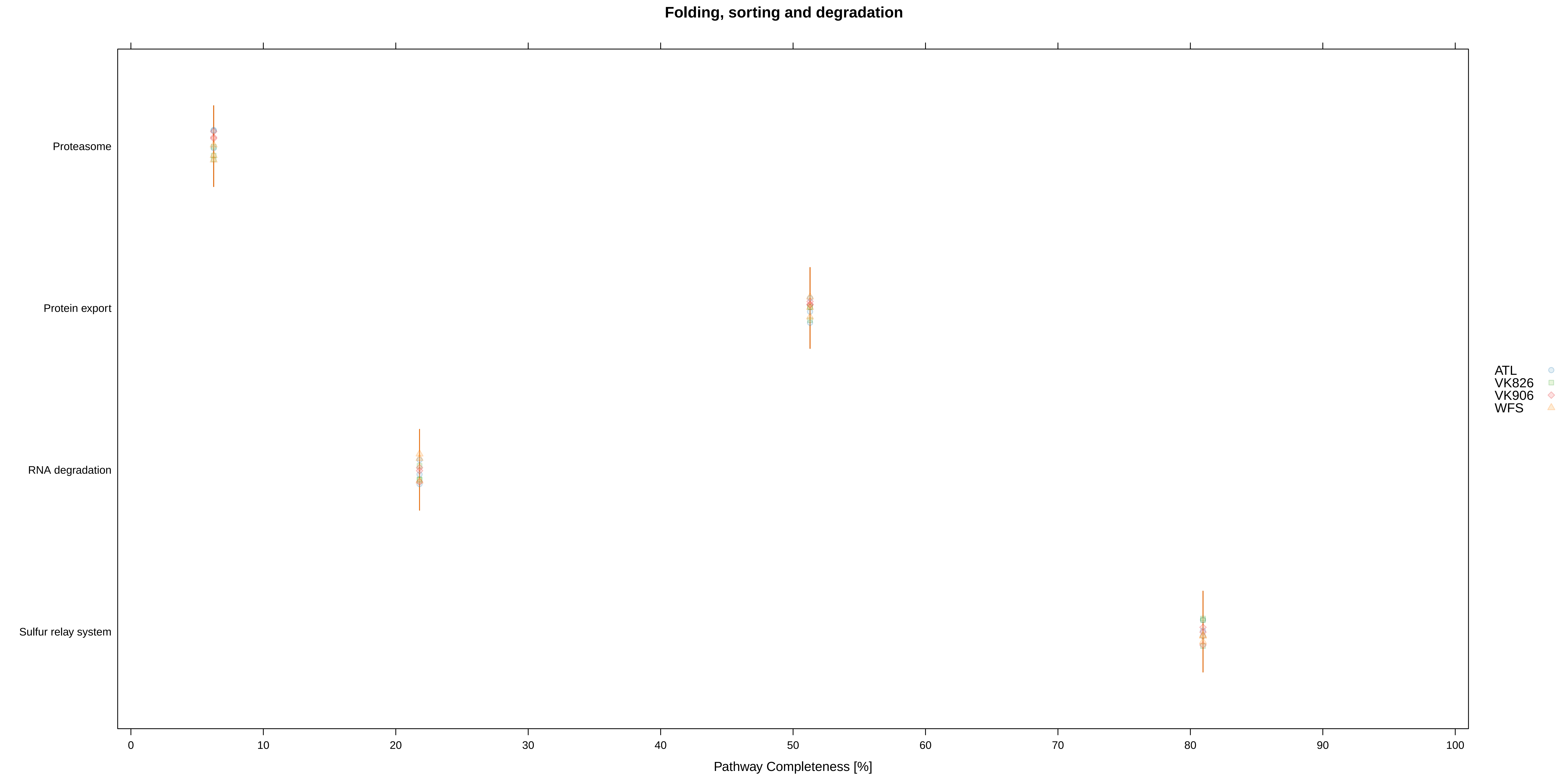
Folding, sorting and degradation
Proteasome
Protein export
RNA degradation
Sulfur relay system
0
10
20
30
40
50
60
70
80
90
100
Pathway Completeness [%]
ATL
VK826
VK906
WFS

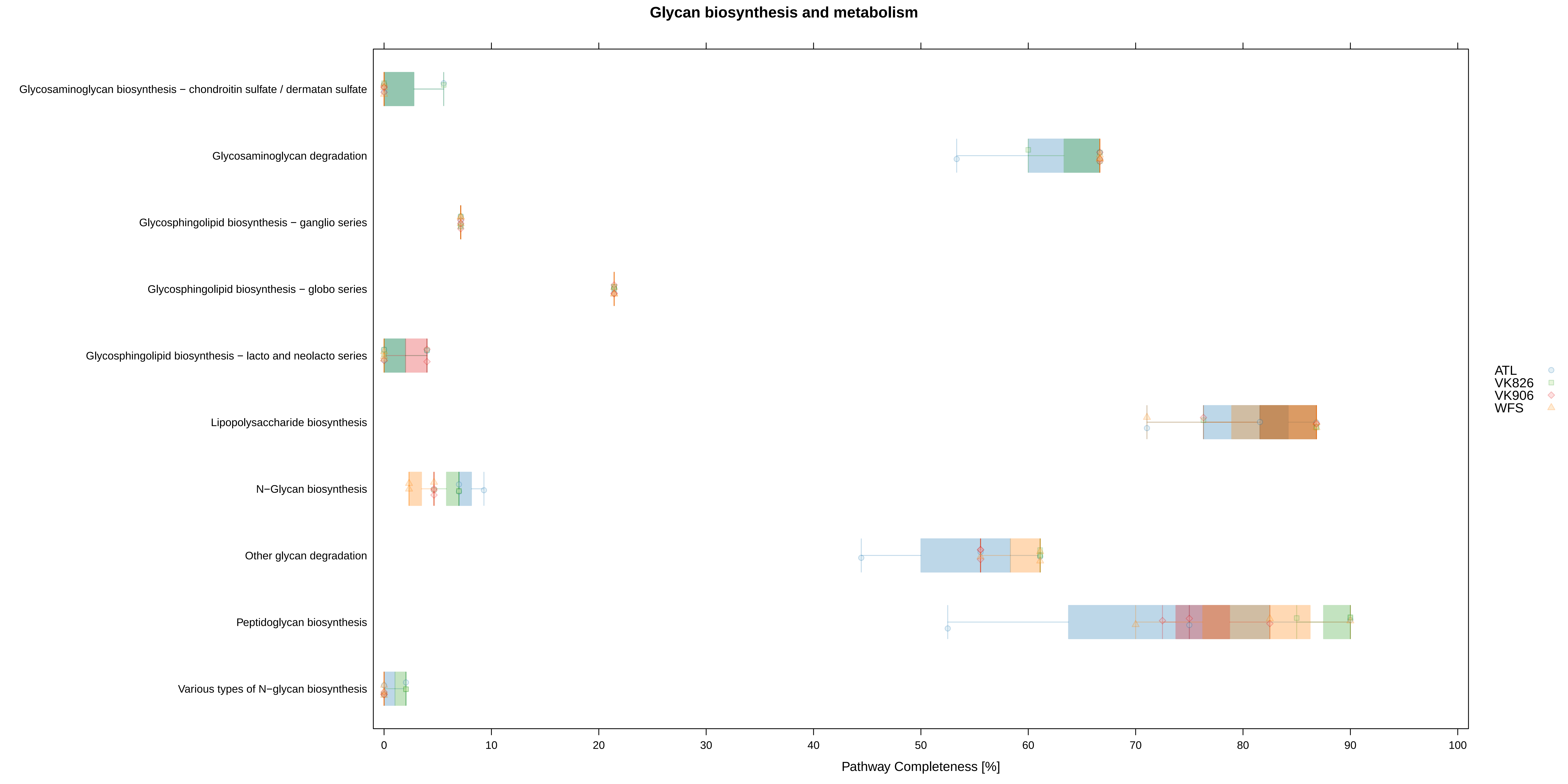

Glycan biosynthesis and metabolism
Glycosaminoglycan biosynthesis − chondroitin sulfate / dermatan sulfate
Glycosaminoglycan degradation
Glycosphingolipid biosynthesis − ganglio series
Glycosphingolipid biosynthesis − globo series
Glycosphingolipid biosynthesis − lacto and neolacto series
Lipopolysaccharide biosynthesis
N−Glycan biosynthesis
Other glycan degradation
Peptidoglycan biosynthesis
Various types of N−glycan biosynthesis
0
10
20
30
40
50
60
70
80
90
100
Pathway Completeness [%]
ATL
VK826
VK906
WFS

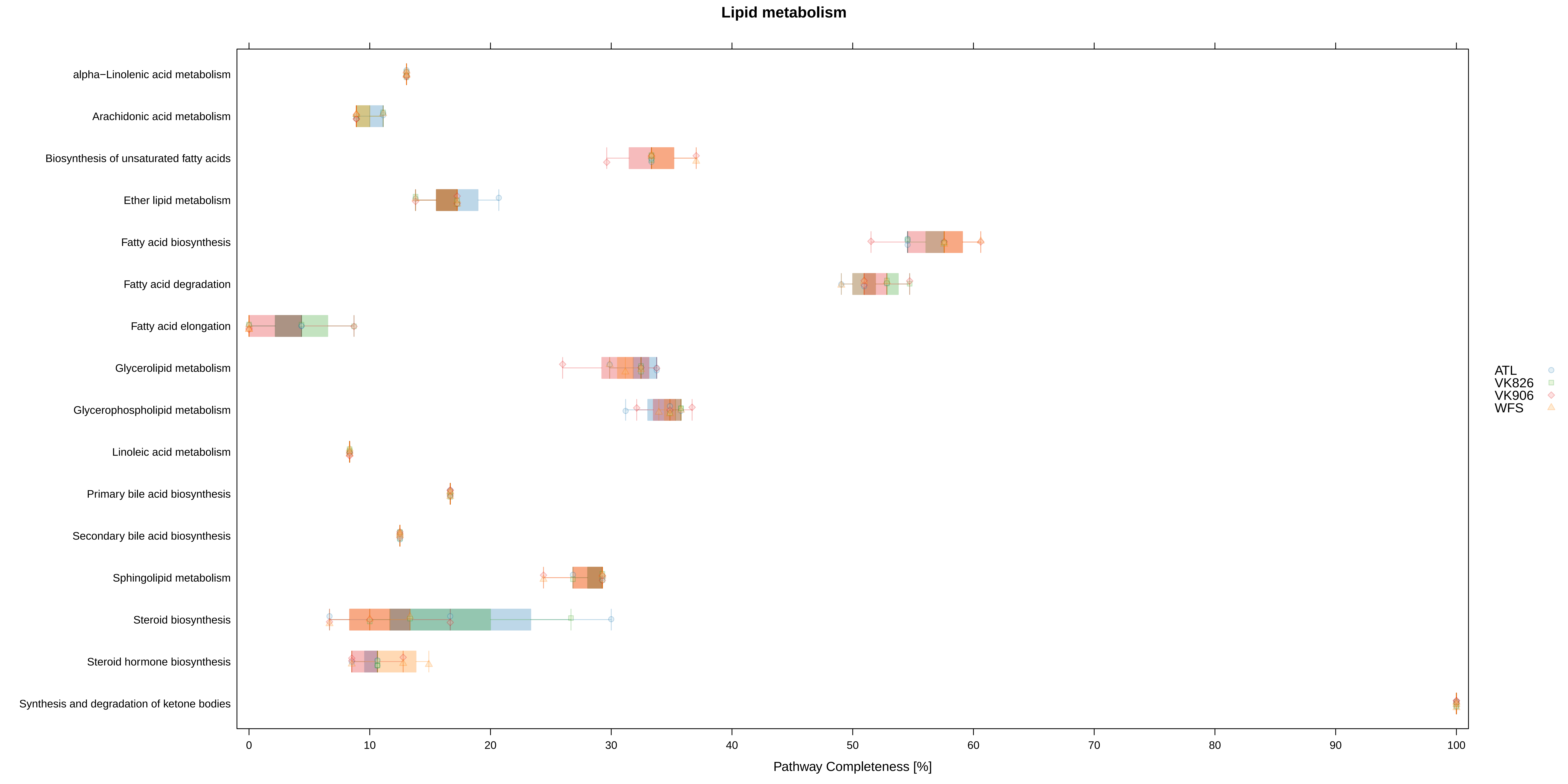

Lipid metabolism
alpha−Linolenic acid metabolism
Arachidonic acid metabolism
Biosynthesis of unsaturated fatty acids
Ether lipid metabolism
Fatty acid biosynthesis
Fatty acid degradation
Fatty acid elongation
Glycerolipid metabolism
Glycerophospholipid metabolism
Linoleic acid metabolism
Primary bile acid biosynthesis
Secondary bile acid biosynthesis
Sphingolipid metabolism
Steroid biosynthesis
Steroid hormone biosynthesis
Synthesis and degradation of ketone bodies
0
10
20
30
40
50
60
70
80
90
100
Pathway Completeness [%]
ATL
VK826
VK906
WFS

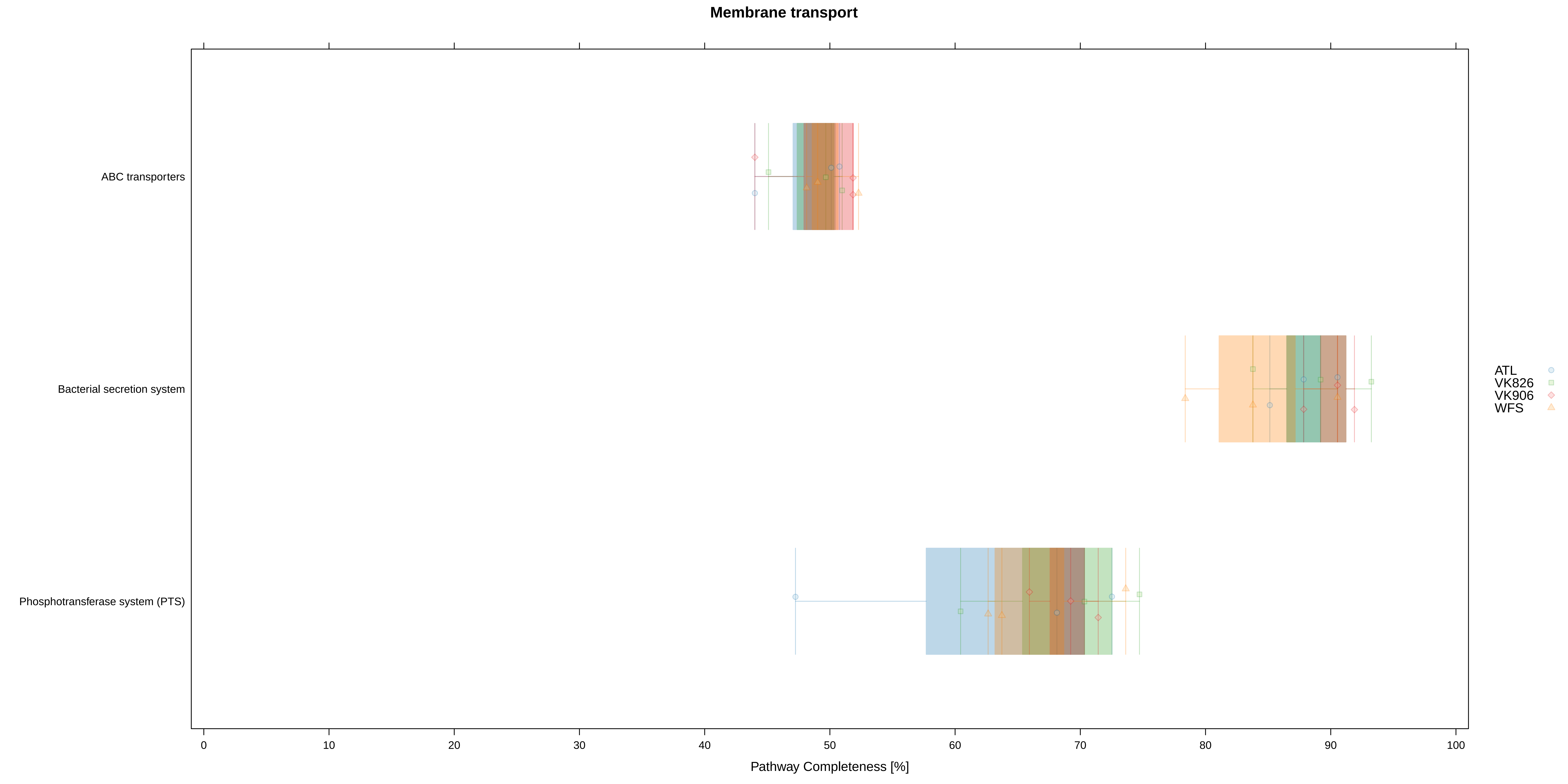
Membrane transport
ABC transporters
Bacterial secretion system
Phosphotransferase system (PTS)
0
10
20
30
40
50
60
70
80
90
100
Pathway Completeness [%]
ATL
VK826
VK906
WFS

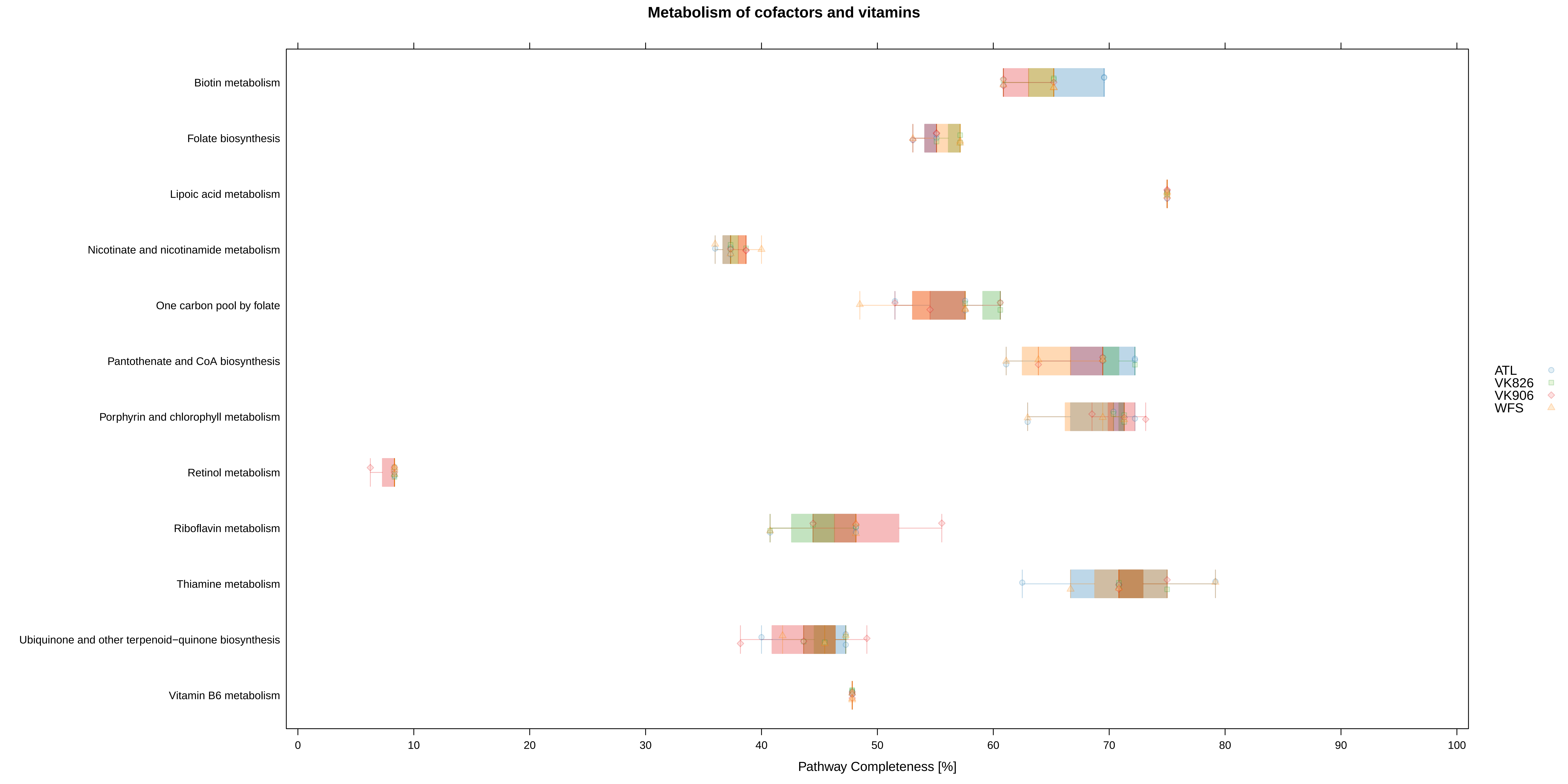

Metabolism of cofactors and vitamins
Biotin metabolism
Folate biosynthesis
Lipoic acid metabolism
Nicotinate and nicotinamide metabolism
One carbon pool by folate
Pantothenate and CoA biosynthesis
Porphyrin and chlorophyll metabolism
Retinol metabolism
Riboflavin metabolism
Thiamine metabolism
Ubiquinone and other terpenoid−quinone biosynthesis
Vitamin B6 metabolism
0
10
20
30
40
50
60
70
80
90
100
Pathway Completeness [%]
ATL
VK826
VK906
WFS

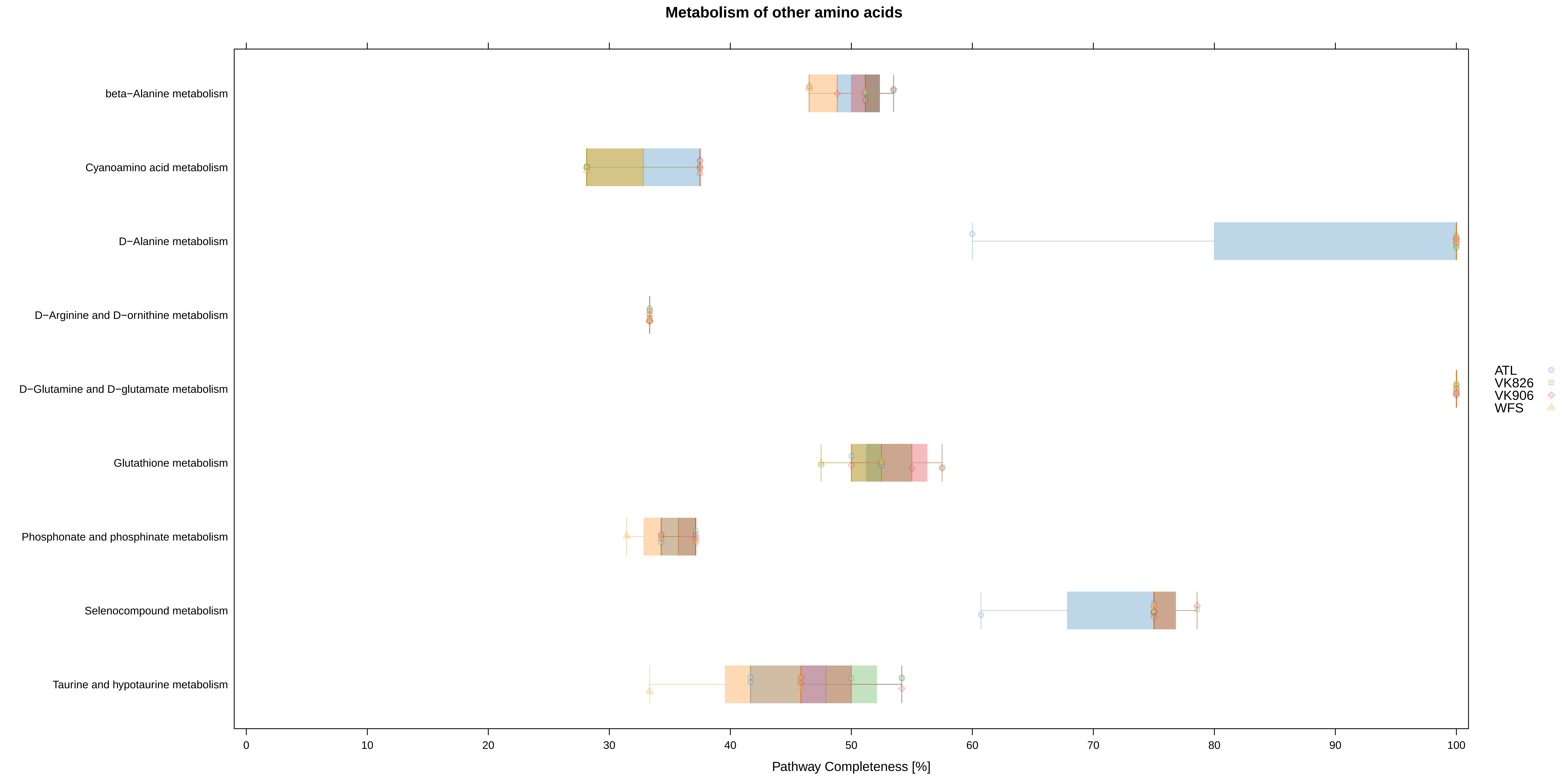
Metabolism of other amino acids
beta−Alanine metabolism
Cyanoamino acid metabolism
D−Alanine metabolism
D−Arginine and D−ornithine metabolism
D−Glutamine and D−glutamate metabolism
Glutathione metabolism
Phosphonate and phosphinate metabolism
Selenocompound metabolism
Taurine and hypotaurine metabolism
0
10
20
30
40
50
60
70
80
90
100
Pathway Completeness [%]
ATL
VK826
VK906
WFS

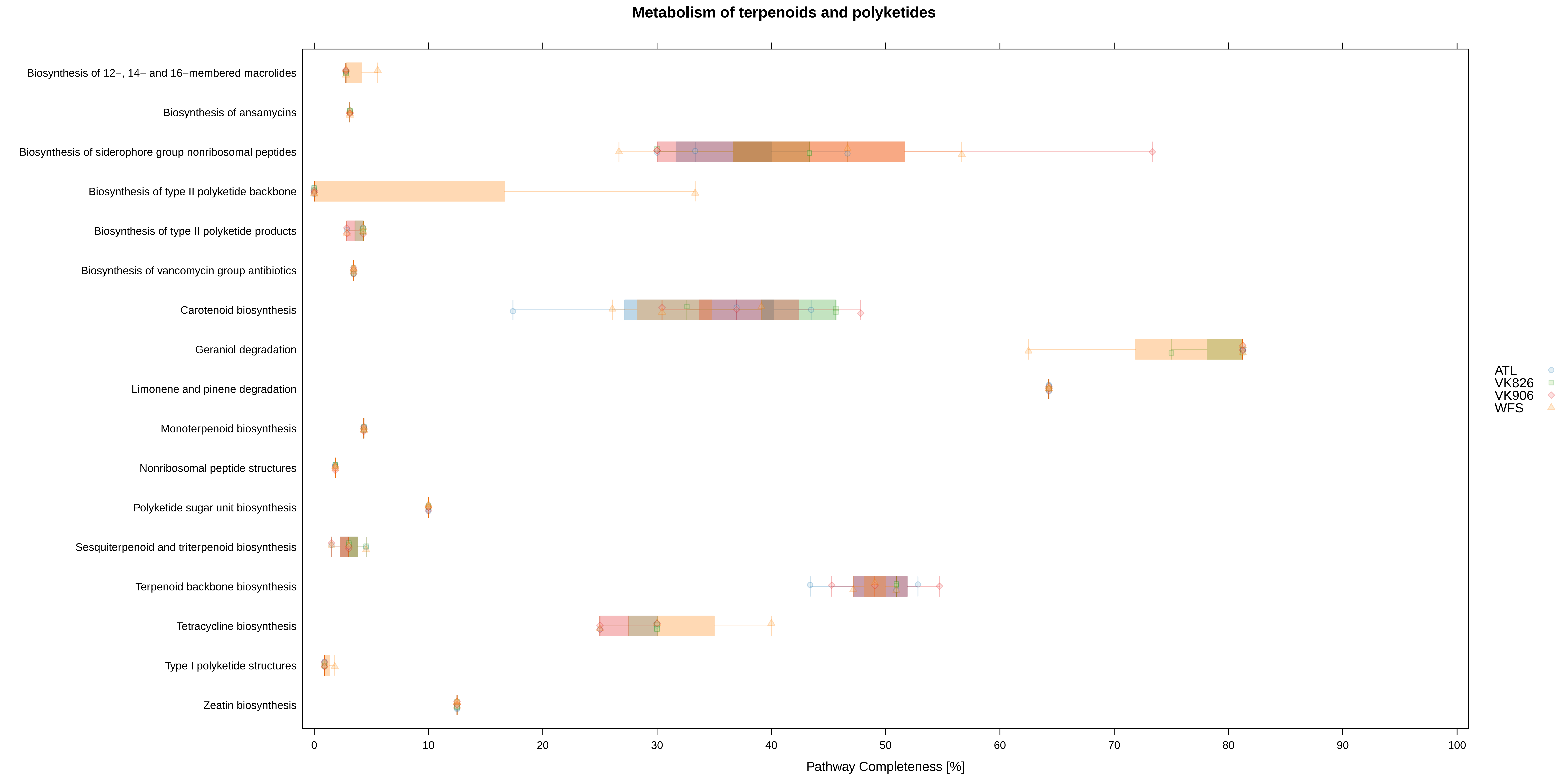
Metabolism of terpenoids and polyketides
Biosynthesis of 12−, 14− and 16−membered macrolides
Biosynthesis of ansamycins
Biosynthesis of siderophore group nonribosomal peptides
Biosynthesis of type II polyketide backbone
Biosynthesis of type II polyketide products
Biosynthesis of vancomycin group antibiotics
Carotenoid biosynthesis
Geraniol degradation
Limonene and pinene degradation
Monoterpenoid biosynthesis
Nonribosomal peptide structures
Polyketide sugar unit biosynthesis
Sesquiterpenoid and triterpenoid biosynthesis
Terpenoid backbone biosynthesis
Tetracycline biosynthesis
Type I polyketide structures
Zeatin biosynthesis
0
10
20
30
40
50
60
70
80
90
100
Pathway Completeness [%]
ATL
VK826
VK906
WFS

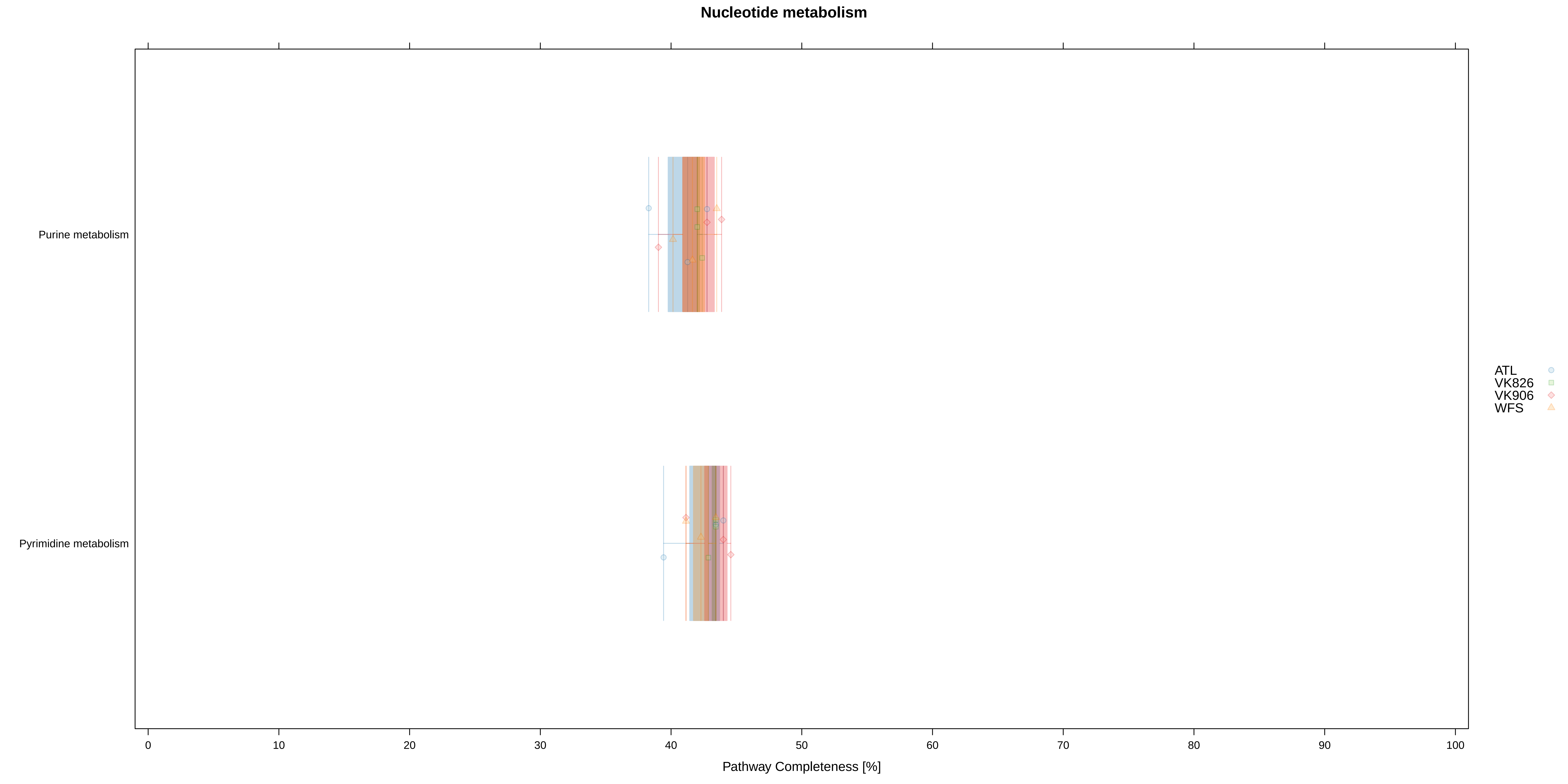
Nucleotide metabolism
Purine metabolism
Pyrimidine metabolism
0
10
20
30
40
50
60
70
80
90
100
Pathway Completeness [%]
ATL
VK826
VK906
WFS

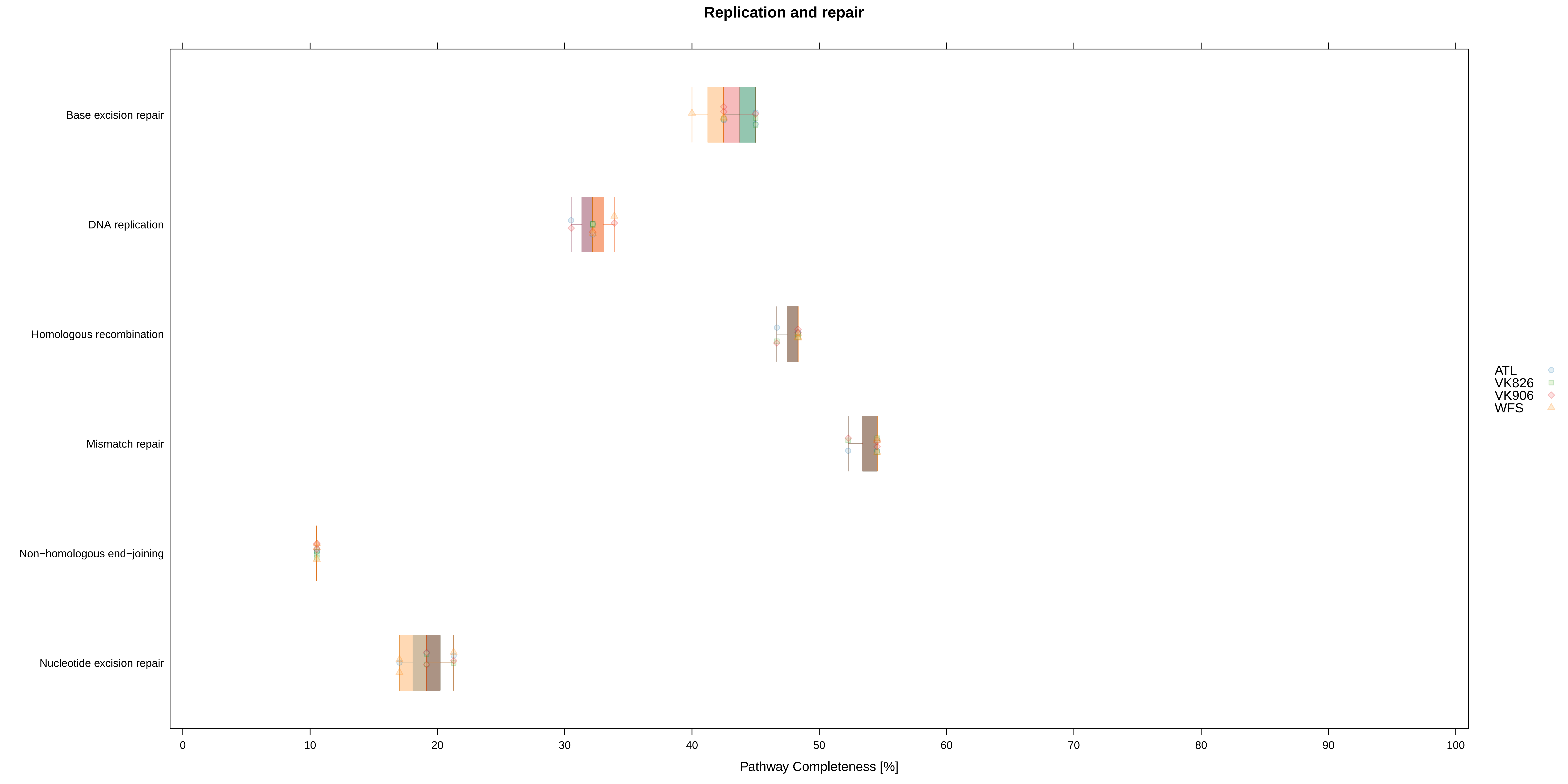
Replication and repair
Base excision repair
DNA replication
Homologous recombination
Mismatch repair
Non−homologous end−joining
Nucleotide excision repair
0
10
20
30
40
50
60
70
80
90
100
Pathway Completeness [%]
ATL
VK826
VK906
WFS

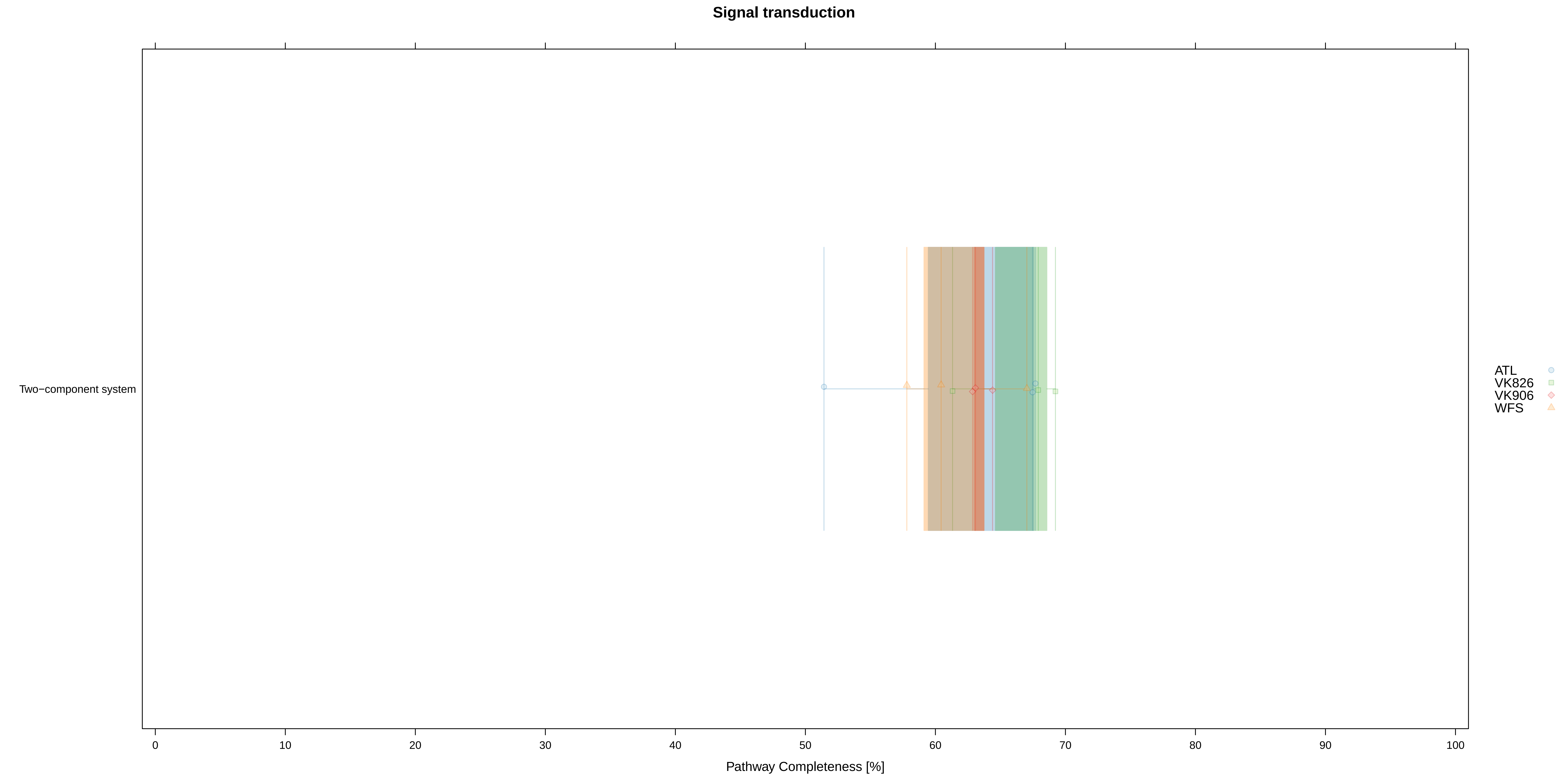
Signal transduction
Two−component system
0
10
20
30
40
50
60
70
80
90
100
Pathway Completeness [%]
ATL
VK826
VK906
WFS

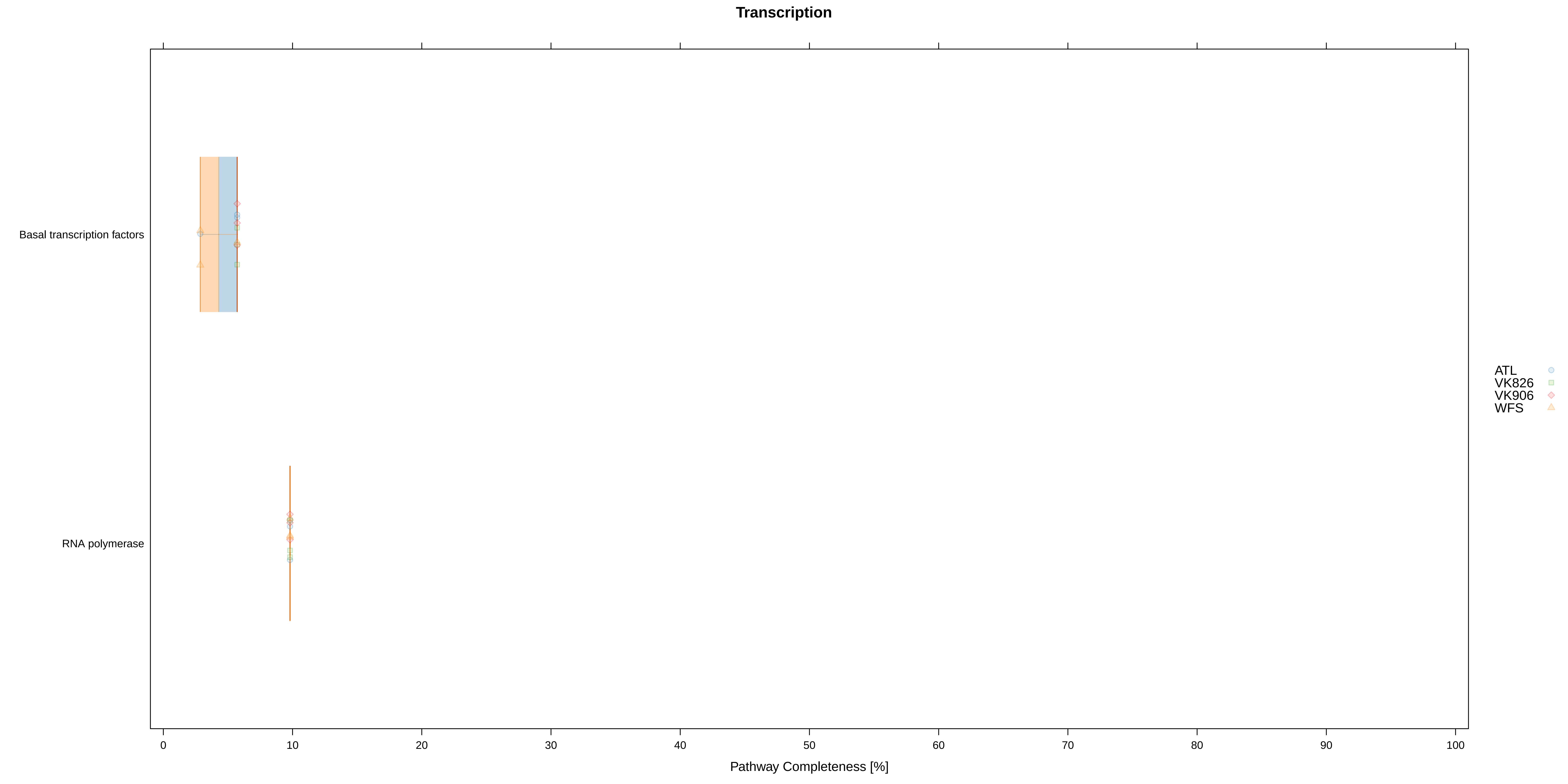
Transcription
Basal transcription factors
RNA polymerase
0
10
20
30
40
50
60
70
80
90
100
Pathway Completeness [%]
ATL
VK826
VK906
WFS

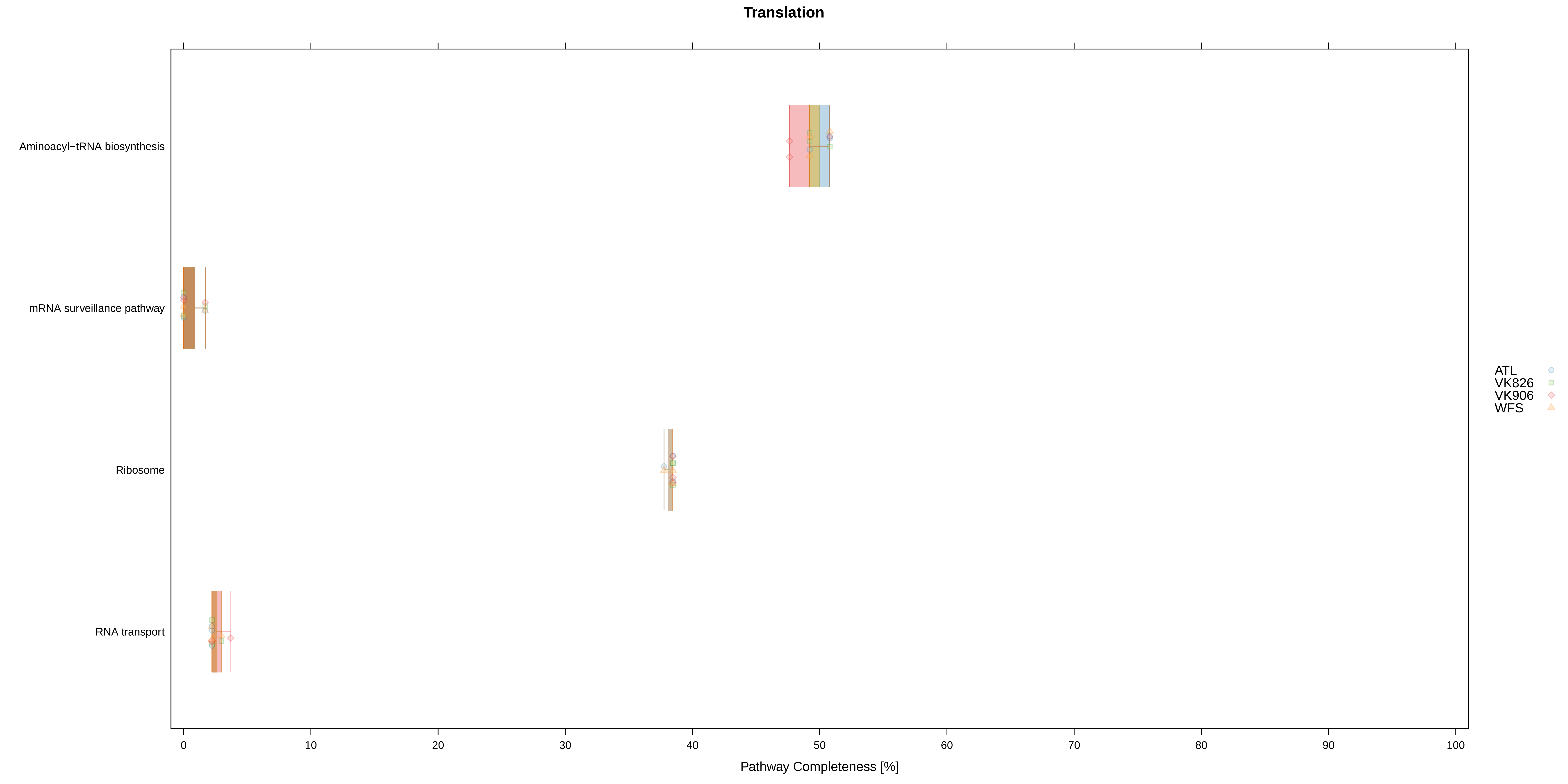
Translation
Aminoacyl−tRNA biosynthesis
mRNA surveillance pathway
Ribosome
RNA transport
0
10
20
30
40
50
60
70
80
90
100
Pathway Completeness [%]
ATL
VK826
VK906
WFS

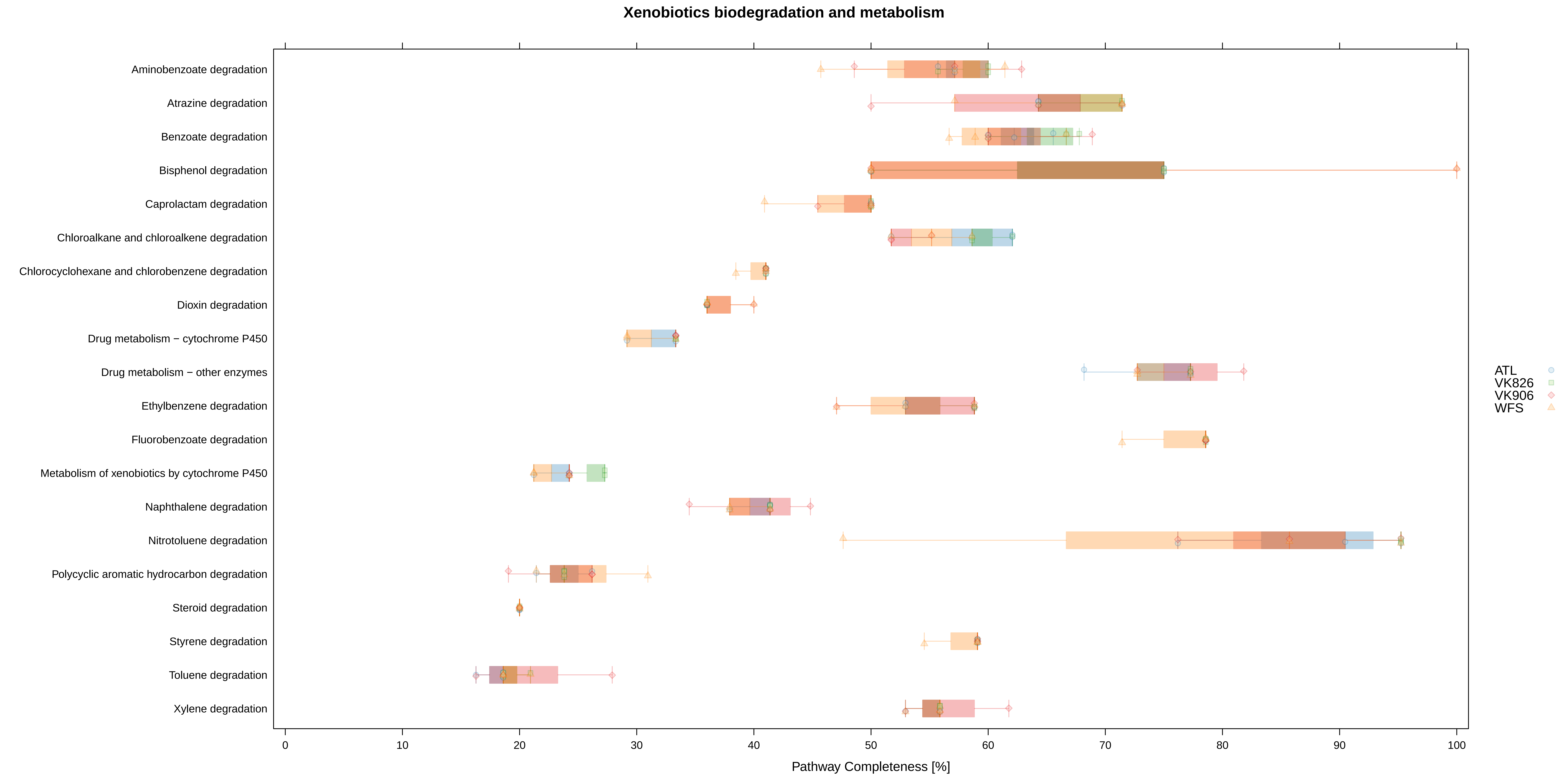

Xenobiotics biodegradation and metabolism
Aminobenzoate degradation
Atrazine degradation
Benzoate degradation
Bisphenol degradation
Caprolactam degradation
Chloroalkane and chloroalkene degradation
Chlorocyclohexane and chlorobenzene degradation
Dioxin degradation
Drug metabolism − cytochrome P450
Drug metabolism − other enzymes
Ethylbenzene degradation
Fluorobenzoate degradation
Metabolism of xenobiotics by cytochrome P450
Naphthalene degradation
Nitrotoluene degradation
Polycyclic aromatic hydrocarbon degradation
Steroid degradation
Styrene degradation
Toluene degradation
Xylene degradation
0
10
20
30
40
50
60
70
80
90
100
Pathway Completeness [%]
ATL
VK826
VK906
WFS
