# Supplementary material for: Biogeographic Comparison of Lophelia-Associated Bacterial Communities in the Western Atlantic Reveals Conserved Core Microbiome
Source: Front Microbiol. 2017 May 4;8:796. doi: 10.3389/fmicb.2017.00796 (PMC5415624; doi:10.3389/fmicb.2017.00796)
Supplement: Supplementary file 1 [file Data_Sheet_1.ZIP › Lophelia_supplementary_material/Lophelia_pathway_phylum_amplicon.pdf]

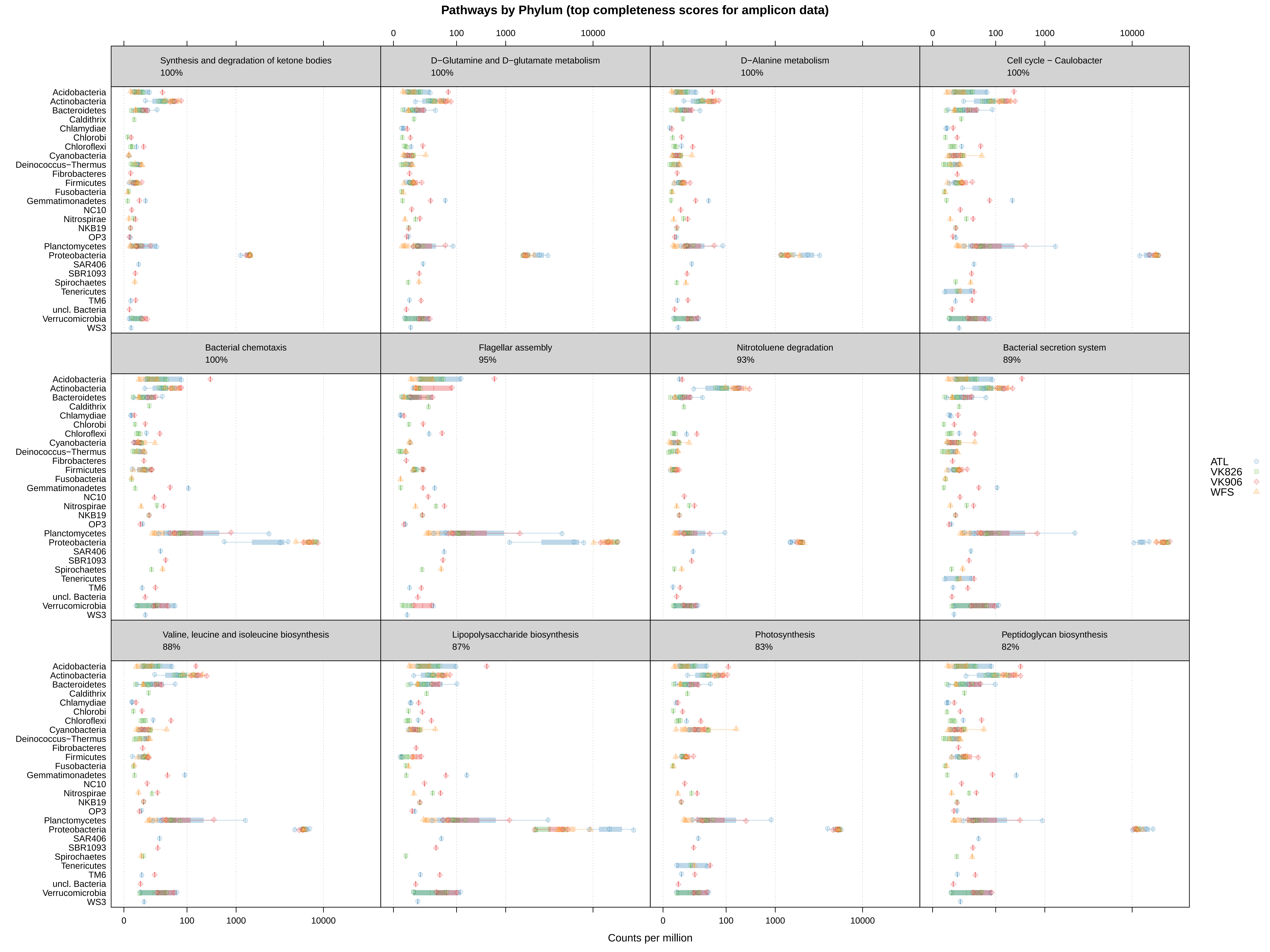

Pathways by Phylum (top completeness scores for amplicon data)
Synthesis and degradation of ketone bodies
100%
D−Glutamine and D−glutamate metabolism
100%
D−Alanine metabolism
100%
Cell cycle − Caulobacter
100%
Bacterial chemotaxis
100%
Flagellar assembly
95%
Nitrotoluene degradation
93%
Bacterial secretion system
89%
Valine, leucine and isoleucine biosynthesis
88%
Lipopolysaccharide biosynthesis
87%
Photosynthesis
83%
Peptidoglycan biosynthesis
82%
Acidobacteria
Actinobacteria
Bacteroidetes
Caldithrix
Chlamydiae
Chlorobi
Chloroflexi
Cyanobacteria
Deinococcus−Thermus
Fibrobacteres
Firmicutes
Fusobacteria
Gemmatimonadetes
NC10
Nitrospirae
NKB19
OP3
Planctomycetes
Proteobacteria
SAR406
SBR1093
Spirochaetes
Tenericutes
TM6
uncl. Bacteria
Verrucomicrobia
WS3
0
100
1000
10000
Counts per million
ATL
VK826
VK906
WFS

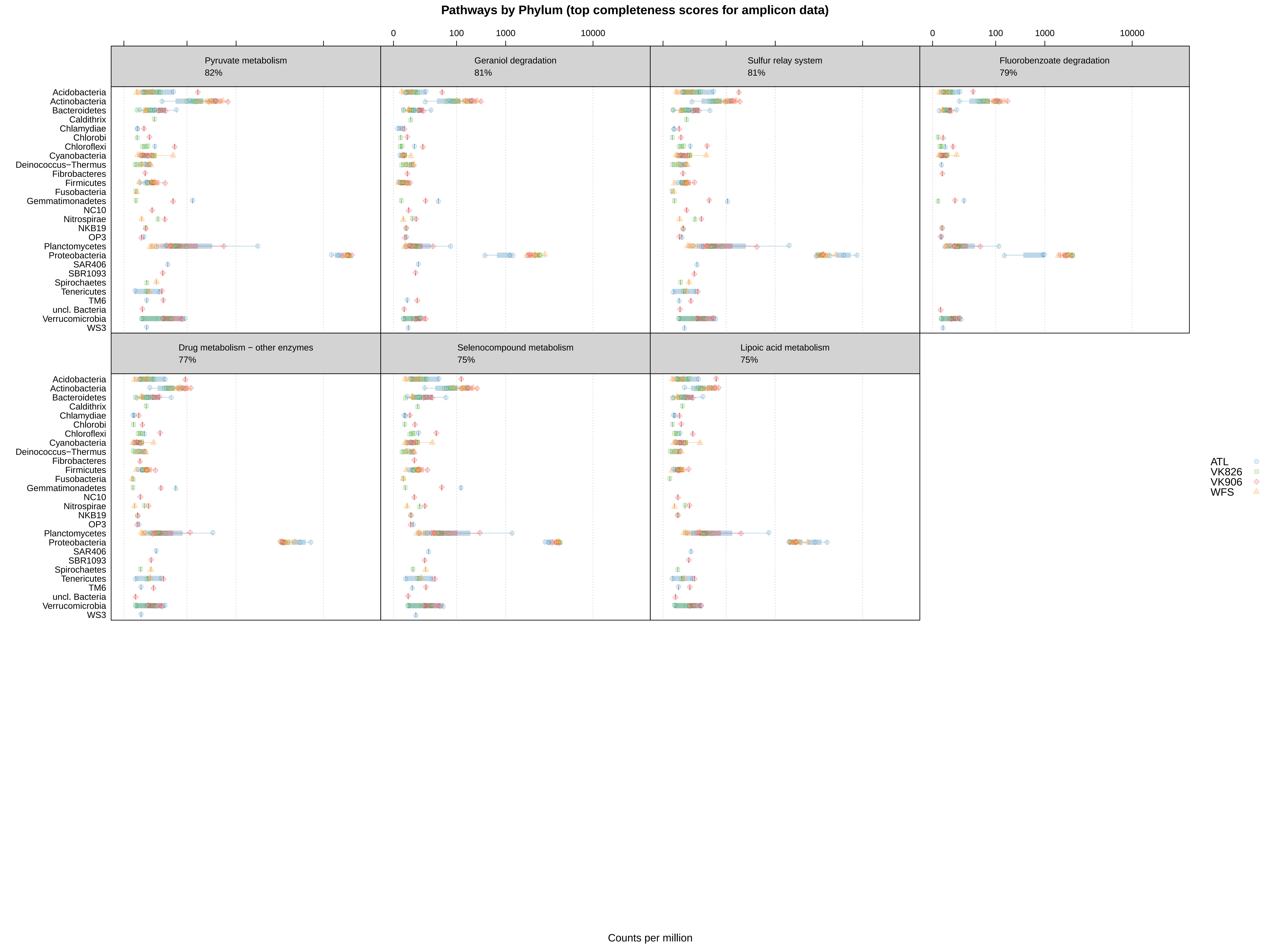
Pathways by Phylum (top completeness scores for amplicon data)
0
100
1000
10000
Pyruvate metabolism
82%
Geraniol degradation
81%
Sulfur relay system
81%
Fluorobenzoate degradation
79%
Drug metabolism − other enzymes
77%
Selenocompound metabolism
75%
Lipoic acid metabolism
75%
Acidobacteria
Actinobacteria
Bacteroidetes
Caldithrix
Chlamydiae
Chlorobi
Chloroflexi
Cyanobacteria
Deinococcus−Thermus
Fibrobacteres
Firmicutes
Fusobacteria
Gemmatimonadetes
NC10
Nitrospirae
NKB19
OP3
Planctomycetes
Proteobacteria
SAR406
SBR1093
Spirochaetes
Tenericutes
TM6
uncl. Bacteria
Verrucomicrobia
WS3
ATL
VK826
VK906
WFS
Counts per million
